# Supplementary material for: A generalized test of genotype–phenotype causality in population-sampled nuclear families
Source: PLoS Genet. 2026 Jul 15;22(7):e1012231. doi: 10.1371/journal.pgen.1012231 (PMC13390957; doi:10.1371/journal.pgen.1012231)
Supplement: S1 Appendix — (PDF) [file pgen.1012231.s001.pdf]

Supporting Information

A generalized test of genotype–phenotype causality in population-sampled nuclear families

Yushi Tang[1] and John D. Storey[1*]

1 Lewis-Sigler Institute for Integrative Genomics, Princeton University, NJ 08544, USA

*Corresponding author: `jstorey@princeton.edu`

Contents

A Data and theory **1**

A.1 Genotype data in a nuclear family 1

A.2 Permutation test 1

A.3 Offspring genotype probabilities 1

A.4 Proof of Theorem 1 3

A.5 Proof of Lemma 1 3

A.6 Proof of Lemma 2 4

A.7 Proof of Theorem 2 7

A.8 Proof of Theorem 3 8

B Simulations **17**

B.1 Simulating genotypes 17

B.2 Simulating quantitative traits 18

B.3 Numerical data underlying Fig 2B 19

B.4 Simulating linkage disequilibrium (LD) 20

B.5 Simulating family-specific confounding effects 22

C UK Biobank data analysis **25**

C.1 Identifying trios 25

C.2 Blood pressure phenotypes quality control 25

C.3 Top significant variants supported by existing studies 25

A Data and theory

A.1 Genotype data in a nuclear family

Here, we describe the data structure of genotypes in a nuclear family. For family j ($j \in [1:J]$) and genetic marker i ($i \in [1:I]$), let $Z^m_{i,j} \in \{0,1,2\}$ be the genotype of the mother, $Z^p_{i,j} \in \{0,1,2\}$ the genotype of the father, and $G_{i,j,k} \in \{0,1,2\}$ the genotype of offspring k for $k \in [1:K_j]$. The families are $j \in [1:J]$ and genetic markers . Fig A depicts a full set of genotypes in a nuclear family, as well as the inheritance process of randomized allele transmissions.

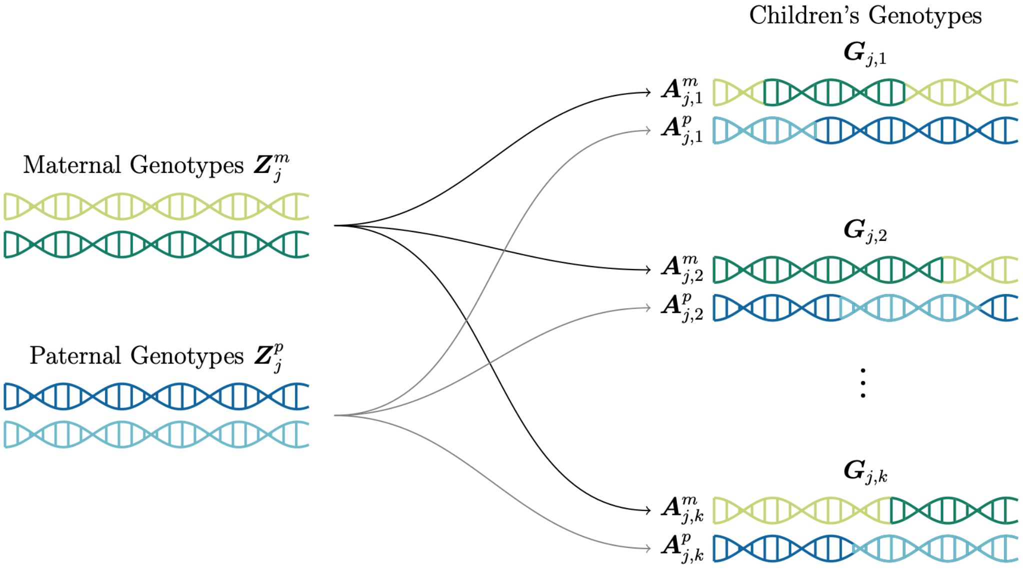

Fig A: **Schematic of randomized genetic transmission in a nuclear family.**

A.2 Permutation test

The permutation test is carried out by permuting the offspring phenotypes over B iterations. In each round of permutation, let $\{Y^*_{j,k}\}$ be the permuted phenotypes We calculate

$$\hat{\mu}_b = \frac{1}{N}\sum_{j=1}^{J}\sum_{k=1}^{K_j} Y^*_{j,k}(W^1_{j,k} + W^0_{j,k}), \qquad d_b = \frac{2}{N}\sum_{j=1}^{J}\sum_{k=1}^{K_j}(W^1_{j,k} - W^0_{j,k})(Y^*_{j,k} - \hat{\mu}_b).$$

We then calculate the permutation p-value by $p_{\text{perm}} = \sum_{b=1}^{B} \mathcal{I}(|d_b| \geq |d_{\text{gTMT}}|)/B$.

A.3 Offspring genotype probabilities

Table A: **Probabilities of observing offspring genotypes given parental genotypes.**

(a) Parent-child trios with one child per family

Offspring Genotype G	Parental Genotypes (Z^m, Z^p)	Conditional Probability $\mathbb{P}(G\|Z^m, Z^p)$
2	(2,2)	1
2	(2,1)	1/2
2	(1,2)	1/2
2	(1,1)	1/4
1	(2,1)	1/2
1	(1,2)	1/2
1	(2,0)	1
1	(0,2)	1
1	(1,1)	1/2
1	(1,0)	1/2
1	(0,1)	1/2
0	(1,1)	1/4
0	(1,0)	1/2
0	(0,1)	1/2
0	(0,0)	1

(b) Parent-offspring tetrads with two offspring per family

Sibling Pairs' Genotypes $(G_{j,k}, G_{j,h})$	Parental Genotypes (Z_j^m, Z_j^p)	Conditional Probability $\mathbb{P}(G_{j,k}, G_{j,h}\|Z_j^m, Z_j^p)$
(2,2)	(2,2)	1
(2,2)	(2,1)	1/4
(2,2)	(1,2)	1/4
(2,2)	(1,1)	1/16
(2,1)	(2,1)	1/4
(2,1)	(1,2)	1/4
(2,1)	(1,1)	1/8
(1,2)	(2,1)	1/4
(1,2)	(1,2)	1/4
(1,2)	(1,1)	1/8
(1,1)	(2,1)	1/4
(1,1)	(1,2)	1/4
(1,1)	(2,0)	1
(1,0)	(1,1)	1/8
(1,0)	(1,0)	1/4
(1,0)	(0,1)	1/4
(0,1)	(1,1)	1/8
(0,1)	(1,0)	1/4
(0,1)	(0,1)	1/4
(0,0)	(1,1)	1/16
(0,0)	(1,0)	1/4
(0,0)	(0,1)	1/4
(0,0)	(0,0)	1

A.4 Proof of Theorem 1

Plugging Eq (11) into δ_{TMT} leads to the following.

$$\begin{aligned}
\delta_{\text{TMT}} &= \frac{1}{2}\mathbb{E}[Y^m_{j,k}(1) - Y^m_{j,k}(0)|Z^m_j = 1, N] + \frac{1}{2}\mathbb{E}[Y^p_{j,k}(1) - Y^p_{j,k}(0)|Z^p_j = 1, N] \\
&= \frac{1}{2}(\alpha_2 - \alpha_1)\mathbb{P}(A^p_{j,k} = 1|Z^m_j = 1, N) + \frac{1}{2}(\alpha_1 - \alpha_0)\mathbb{P}(A^p_{j,k} = 0|Z^m_j = 1, N) \\
&\quad + \frac{1}{2}(\alpha_2 - \alpha_1)\mathbb{P}(A^m_{j,k} = 1|Z^p_j = 1, N) + \frac{1}{2}(\alpha_1 - \alpha_0)\mathbb{P}(A^m_{j,k} = 0|Z^p_j = 1, N).
\end{aligned}$$

In practice, $0 < \mathbb{P}(A^m_{j,k} = 1|Z^p_j = 1, N) < 1$ and $0 < \mathbb{P}(A^p_{j,k} = 1|Z^m_j = 1, N) < 1$. Under Assumption 1, either $\alpha_0 \le \alpha_1 \le \alpha_2$ or $\alpha_0 \ge \alpha_1 \ge \alpha_2$. Then $\delta_{\text{TMT}} = 0$ if and only if $\alpha_0 = \alpha_1 = \alpha_2$, which implies $\text{ACE}(G \to Y) = 0$ by Definition 4.

A.5 Proof of Lemma 1

We show on the maternal side $Y^m_{j,k}(0), Y^m_{j,k}(1) \perp\!\!\!\perp A^m_j \;\big|\; Z^m_j = 1$. The proof for the paternal side follows the same strategy. We first show that

$$A^m_{j,k} \perp\!\!\!\perp A^p_{j,k}|Z^m_j = 1, \tag{S1}$$

which is equivalent to prove

$$\mathbb{P}(A^p_{j,k} = a|A^m_{j,k} = b, Z^m_j = 1) = \mathbb{P}(A^p_{j,k} = a|Z^m_j = 1) \tag{S2}$$

for $a, b \in \{0, 1\}$. First, we directly calculate the following conditional covariance:

$$\begin{aligned}
&\mathbb{C}(A^m_{j,k}, A^p_{j,k}|Z^m_j = 1) \\
&= \mathbb{E}[A^m_{j,k}A^p_{j,k}|Z^m_j = 1] - \mathbb{E}[A^m_{j,k}|Z^m_j = 1]\mathbb{E}[A^p_{j,k}|Z^m_j = 1] \\
&= \mathbb{P}(A^m_{j,k} = 1, A^p_{j,k} = 1|Z^m_j = 1) - \mathbb{P}(A^m_{j,k} = 1|Z^m_j = 1)\mathbb{P}(A^p_{j,k} = 1|Z^m_j = 1) \\
&= \mathbb{P}(A^m_{j,k} = 1|Z^m_j = 1)\mathbb{P}(A^p_{j,k} = 1|A^m_{j,k} = 1, Z^m_j = 1) - \frac{1}{2}\mathbb{P}(A^p_{j,k} = 1|Z^m_j = 1) \\
&= \frac{1}{2}\mathbb{P}(A^p_{j,k} = 1|A^m_{j,k} = 1, Z^m_j = 1) - \frac{1}{2}\mathbb{P}(A^p_{j,k} = 1|Z^m_j = 1).
\end{aligned} \tag{S3}$$

By the Law of Total Covariance,

$$\begin{aligned}
&\mathbb{C}(A^m_{j,k}, A^p_{j,k}|Z^m_j = 1) \\
&= \mathbb{E}\Big[\mathbb{C}(A^m_{j,k}, A^p_{j,k}|Z^m_j = 1, Z^p_j = 1)\Big|Z^m_j = 1\Big] \\
&\qquad + \mathbb{C}\Big(\mathbb{E}[A^m_{j,k}|Z^m_j = 1, Z^p_j = 1], \mathbb{E}[A^p_{j,k}|Z^m_j = 1, Z^p_j = 1]\Big|Z^m_j = 1\Big)
\end{aligned}$$

$$= \mathbb{E}\Big[\mathbb{E}[A^m_{j,k}A^p_{j,k}|Z^m_j = 1, Z^p_j = 1] - \mathbb{E}[A^m_{j,k}|Z^m_j = 1, Z^p_j = 1]\mathbb{E}[A^p_{j,k}|Z^m_j = 1, Z^p_j = 1]\Big|Z^m_j = 1\Big]$$
$$+ \underbrace{\mathbb{C}\Big(\frac{1}{2}, \frac{1}{2}\Big|Z^m_j = 1\Big)}_{=0}$$
$$= \mathbb{E}\Big[\frac{1}{4} - \frac{1}{2} \times \frac{1}{2}\Big|Z^m_j = 1\Big]$$
$$= 0. \tag{S4}$$

Since Eq (S3) and Eq (S4) are equal, this implies $\mathbb{P}(A^p_{j,k} = 1|A^m_{j,k} = 1, Z^m_j = 1) = \mathbb{P}(A^p_{j,k} = 1|Z^m_j = 1)$. Similarly, $\mathbb{P}(A^p_{j,k} = a|A^m_{j,k} = b, Z^m_j = 1) = \mathbb{P}(A^p_{j,k} = a|Z^m_j = 1)$ for $a, b \in \{0, 1\}$. Therefore, $A^m_{j,k} \perp\!\!\!\perp A^p_{j,k}|Z^m_j = 1$. By Assumption 2, $\epsilon_{j,k}$ and γ_j are also independent of $A^m_{j,k}$ conditional on $Z^m_j = 1$. Thus, all random variables contributing to $Y^m_{j,k}(0)$ and $Y^m_{j,k}(1)$ in Eq (10) are independent of A^m_j conditional on $Z^m_j = 1$, which completes the proof.

A.6 Proof of Lemma 2

We first show that $\mathbb{E}\big[(W^1_{j,k} - W^0_{j,k})\hat{\mu}\big|N\big] = 0$. We write $\hat{\mu}$ in Eq (4) as $\hat{\mu} = \frac{1}{N}\sum_{l=1}^{J}\sum_{s=1}^{K_l}(W^0_{l,s} + W^1_{l,s})Y_{l,s}$. Then

$$\mathbb{E}\big[(W^1_{j,k} - W^0_{j,k})\hat{\mu}\big|N\big]$$
$$= \mathbb{E}\left[(W^1_{j,k} - W^0_{j,k})\frac{1}{N}\sum_{l=1}^{J}\sum_{s=1}^{K_l}(W^0_{l,s} + W^1_{l,s})Y_{l,s}\middle| N\right]$$
$$= \frac{1}{N}\mathbb{E}\left[(W^1_{j,k} - W^0_{j,k})\sum_{l=1}^{J}\sum_{s=1}^{K_l}(W^0_{l,s} + W^1_{l,s})(Y_{l,s} - \epsilon_{l,s} - \gamma_l)\middle| N\right] \tag{S5}$$
$$+ \frac{1}{N}\mathbb{E}\left[(W^1_{j,k} - W^0_{j,k})\sum_{l=1}^{J}\sum_{s=1}^{K_l}(W^0_{l,s} + W^1_{l,s})(\epsilon_{l,s} + \gamma_l)\middle| N\right]. \tag{S6}$$

To show that Eq (S5) equals 0, define

$$N_0 = \sum_{l=1}^{J}\sum_{s=1}^{K_l}\mathcal{I}(G_{l,s} = 0)\big(\mathcal{I}(Z^m_l = 1) + \mathcal{I}(Z^p_l = 1)\big),$$
$$N_1 = \sum_{l=1}^{J}\sum_{s=1}^{K_l}\mathcal{I}(G_{l,s} = 1)\big(\mathcal{I}(Z^m_l = 1) + \mathcal{I}(Z^p_l = 1)\big),$$
$$N_2 = \sum_{l=1}^{J}\sum_{s=1}^{K_l}\mathcal{I}(G_{l,s} = 2)\big(\mathcal{I}(Z^m_l = 1) + \mathcal{I}(Z^p_l = 1)\big).$$

Also observe that

$$W^0_{l,s} + W^1_{l,s} = \mathcal{I}(Z^m_l = 1) + \mathcal{I}(Z^p_l = 1).$$

We use the above equations for N_0, N_1, N_2, and $W^0_{l,s} + W^1_{l,s}$. We also recall the trait model in Eq (9) to derive Eq (S5) as

$$\begin{aligned}
&\frac{1}{N}\mathbb{E}\left[(W^1_{j,k} - W^0_{j,k})\sum_{l=1}^{J}\sum_{s=1}^{K_l}\big(\mathcal{I}(Z^m_l = 1) + \mathcal{I}(Z^p_l = 1)\big)(Y_{l,s} - \epsilon_{l,s} - \gamma_l)\middle| N\right] \\
&= \frac{1}{N}\mathbb{E}\left[(W^1_{j,k} - W^0_{j,k})\sum_{l=1}^{J}\sum_{s=1}^{K_l}\begin{pmatrix} \alpha_0\mathcal{I}(G_{l,s} = 0)\big(\mathcal{I}(Z^m_l = 1) + \mathcal{I}(Z^p_l = 1)\big) \\ +\alpha_1\mathcal{I}(G_{l,s} = 1)\big(\mathcal{I}(Z^m_l = 1) + \mathcal{I}(Z^p_l = 1)\big) \\ +\alpha_2\mathcal{I}(G_{l,s} = 2)\big(\mathcal{I}(Z^m_l = 1) + \mathcal{I}(Z^p_l = 1)\big)\end{pmatrix}\middle| N\right] \\
&= \frac{1}{N}\mathbb{E}\Big[(W^1_{j,k} - W^0_{j,k})(\alpha_0 N_0 + \alpha_1 N_1 + \alpha_2 N_2)\Big| N\Big] \\
&= \frac{1}{N}\mathbb{E}\Big[\mathbb{E}[(W^1_{j,k} - W^0_{j,k})(\alpha_0 N_0 + \alpha_1 N_1 + \alpha_2 N_2)|N_0, N_1, N_2, N]\Big| N\Big] \\
&= \frac{1}{N}\mathbb{E}\Big[\mathbb{E}[W^1_{j,k} - W^0_{j,k}|N_0, N_1, N_2, N] \times (\alpha_0 N_0 + \alpha_1 N_1 + \alpha_2 N_2)\Big| N\Big].
\end{aligned}$$

Note that

$$\begin{aligned}
&\mathbb{E}[W^1_{j,k} - W^0_{j,k}|N_0, N_1, N_2, N] \\
&= \mathbb{E}\Big[(2A^m_{j,k} - 1)\mathcal{I}(Z^m_j = 1) + (2A^p_{j,k} - 1)\mathcal{I}(Z^p_j = 1)\Big| N_0, N_1, N_2, N\Big] \\
&= \underbrace{\mathbb{E}\left[2A^m_{j,k} - 1 \middle| \begin{array}{c} Z^m_j = 1, Z^p_j \neq 1 \\ N_0, N_1, N_2, N \end{array}\right]}_{=0} \times\mathbb{P}\left(\begin{array}{c} Z^m_j = 1 \\ Z^p_j \neq 1 \end{array}\middle| \begin{array}{c} N_0, N_1 \\ N_2, N \end{array}\right) \\
&\quad + \underbrace{\mathbb{E}\left[2A^p_{j,k} - 1 \middle| \begin{array}{c} Z^m_j \neq 1, Z^p_j = 1 \\ N_0, N_1, N_2, N \end{array}\right]}_{=0} \times\mathbb{P}\left(\begin{array}{c} Z^m_j \neq 1 \\ Z^p_j = 1 \end{array}\middle| \begin{array}{c} N_0, N_1 \\ N_2, N \end{array}\right) \\
&\quad + \underbrace{\mathbb{E}\left[2A^m_{j,k} + 2A^p_{j,k} - 2 \middle| \begin{array}{c} Z^m_j = 1, Z^p_j = 1 \\ N_0, N_1, N_2, N \end{array}\right]}_{=0} \times\mathbb{P}\left(\begin{array}{c} Z^m_j = 1 \\ Z^p_j = 1 \end{array}\middle| \begin{array}{c} N_0, N_1 \\ N_2, N \end{array}\right) \\
&= 0,
\end{aligned}$$

and therefore,

$$(\text{S5}) = \frac{1}{N}\mathbb{E}\Big[0 \times (\alpha_0 N_0 + \alpha_1 N_1 + \alpha_2 N_2)\Big| N\Big] = 0.$$

For Eq (S6),

$$
\begin{aligned}
\text{(S6)} &= \frac{1}{N}\mathbb{E}\left[(W^1_{j,k} - W^0_{j,k}) \sum_{l=1}^{J}\sum_{s=1}^{K_l} \big(\mathcal{I}(Z^m_l = 1) + \mathcal{I}(Z^p_l = 1)\big)(\epsilon_{l,s} + \gamma_l) \middle| N \right] \\
&= \frac{1}{N}\mathbb{E}\left[\left(\begin{array}{c} (2A^m_{j,k} - 1)\mathcal{I}(Z^m_j = 1) \\ +(2A^p_{j,k} - 1)\mathcal{I}(Z^p_j = 1) \end{array} \right) \sum_{l=1}^{J}\sum_{s=1}^{K_l} \left(\begin{array}{c} \mathcal{I}(Z^m_l = 1)(\epsilon_{l,s} + \gamma_l) \\ +\mathcal{I}(Z^p_l = 1)(\epsilon_{l,s} + \gamma_l) \end{array} \right) \middle| N \right] \\
&= \frac{1}{N}\sum_{l=1}^{J}\sum_{s=1}^{K_l}\mathbb{E}\left[\left(\begin{array}{c} (2A^m_{j,k} - 1)\mathcal{I}(Z^m_j = 1) \\ +(2A^p_{j,k} - 1)\mathcal{I}(Z^p_j = 1) \end{array} \right) \left(\begin{array}{c} \mathcal{I}(Z^m_l = 1)(\epsilon_{l,s} + \gamma_l) \\ +\mathcal{I}(Z^p_l = 1)(\epsilon_{l,s} + \gamma_l) \end{array} \right) \middle| N \right] \\
&= \frac{1}{N}\sum_{l=1}^{J}\sum_{s=1}^{K_l}\mathbb{E}\left[(2A^m_{j,k} - 1)(\epsilon_{l,s} + \gamma_l)\mathcal{I}(Z^m_j = 1, Z^m_l = 1) \middle| N \right] \qquad \text{(S7)} \\
&\quad + \frac{1}{N}\sum_{l=1}^{J}\sum_{s=1}^{K_l}\mathbb{E}\left[(2A^m_{j,k} - 1)(\epsilon_{l,s} + \gamma_l)\mathcal{I}(Z^m_j = 1, Z^p_l = 1) \middle| N \right] \\
&\quad + \frac{1}{N}\sum_{l=1}^{J}\sum_{s=1}^{K_l}\mathbb{E}\left[(2A^p_{j,k} - 1)(\epsilon_{l,s} + \gamma_l)\mathcal{I}(Z^p_j = 1, Z^m_l = 1) \middle| N \right] \\
&\quad + \frac{1}{N}\sum_{l=1}^{J}\sum_{s=1}^{K_l}\mathbb{E}\left[(2A^p_{j,k} - 1)(\epsilon_{l,s} + \gamma_l)\mathcal{I}(Z^p_j = 1, Z^p_l = 1) \middle| N \right].
\end{aligned}
$$

Now we show Line (S7) equals 0, and then the three lines that follow equal 0 by the same proof. By Mendel's Law of Segregation,

$$
\begin{aligned}
\mathbb{P}(A^m_{j,k} = 1 | Z^m_j = 1, Z^m_l = 1, N, \gamma_l, \epsilon_{l,s}) = \frac{1}{2} = \mathbb{P}(A^m_{j,k} = 1 | Z^m_j = 1, Z^m_l = 1, N), \\
\mathbb{P}(A^m_{j,k} = 0 | Z^m_j = 1, Z^m_l = 1, N, \gamma_l, \epsilon_{l,s}) = \frac{1}{2} = \mathbb{P}(A^m_{j,k} = 0 | Z^m_j = 1, Z^m_l = 1, N).
\end{aligned}
$$

Then $\mathbb{E}[2A^m_{j,k} - 1 | Z^m_j = 1, Z^m_l = 1, N] = 0$ and $(\epsilon_{l,s}, \gamma_l) \perp\!\!\!\perp A^m_{j,k} | (Z^m_j = 1, Z^m_l = 1, N)$ so that

$$
\begin{aligned}
\text{(S7)} &= \frac{1}{N}\sum_{l=1}^{J}\sum_{s=1}^{K_l}\mathbb{E}\left[(2A^m_{j,k} - 1)(\epsilon_{l,s} + \gamma_l) \middle| \begin{array}{l} Z^m_j = 1 \\ Z^m_l = 1, N \end{array} \right] \times \mathbb{P}\left(\begin{array}{l} Z^m_j = 1 \\ Z^m_l = 1 \end{array} \middle| N \right) \\
&= \frac{1}{N}\sum_{l=1}^{J}\sum_{s=1}^{K_l} \underbrace{\mathbb{E}[2A^m_{j,k} - 1 | Z^m_j = 1, Z^m_l = 1, N]}_{=0} \\
&\qquad \times \mathbb{E}[\epsilon_{l,s} + \gamma_l | Z^m_j = 1, Z^m_l = 1, N] \times \mathbb{P}(Z^m_j = 1, Z^m_l = 1 | N) \\
&= 0.
\end{aligned}
$$

Eq (S6) equals 0 and

$$
\mathbb{E}\big[(W^1_{j,k} - W^0_{j,k})\hat{\mu} \big| N\big] = \text{(S5)} + \text{(S6)} = 0. \qquad \text{(S8)}
$$

Therefore,

$$\mathbb{E}\left[\frac{2}{N}\sum_{j=1}^{J}\sum_{k=1}^{K_j}(W^1_{j,k}-W^0_{j,k})\hat{\mu}\middle|N\right]=\frac{2}{N}\sum_{j=1}^{J}\sum_{k=1}^{K_j}\mathbb{E}\big[(W^1_{j,k}-W^0_{j,k})\hat{\mu}\big|N\big]=0,$$

and

$$\begin{aligned}
\mathbb{E}\left[d_{\mathrm{gTMT}}\middle|N\right]&=\mathbb{E}\left[\frac{2}{N}\sum_{j=1}^{J}\sum_{k=1}^{K_j}(W^1_{j,k}-W^0_{j,k})(Y_{j,k}-\hat{\mu})\middle|N\right]\\
&=\mathbb{E}\left[\frac{2}{N}\sum_{j=1}^{J}\sum_{k=1}^{K_j}(W^1_{j,k}-W^0_{j,k})Y_{j,k}\middle|N\right]-\underbrace{\mathbb{E}\left[\frac{2}{N}\sum_{j=1}^{J}\sum_{k=1}^{K_j}(W^1_{j,k}-W^0_{j,k})\hat{\mu}\middle|N\right]}_{=0}\\
&=\mathbb{E}\left[\frac{2}{N}\sum_{j=1}^{J}\sum_{k=1}^{K_j}(W^1_{j,k}-W^0_{j,k})Y_{j,k}\middle|N\right].
\end{aligned}$$

A.7 Proof of Theorem 2

Let $\mathcal{J}$ be the total number of offspring in that $\mathcal{J}=\sum_{j=1}^{J}K_j$.

$$\begin{aligned}
\mathbb{E}[d_{\mathrm{gTMT}}|N]&=\mathbb{E}\left[\frac{2}{N}\sum_{j=1}^{J}\sum_{k=1}^{K_j}(W^1_{j,k}-W^0_{j,k})(Y_{j,k}-\hat{\mu})\middle|N\right]\\
&=\mathbb{E}\left[\frac{2}{N}\sum_{j=1}^{J}\sum_{k=1}^{K_j}(W^1_{j,k}-W^0_{j,k})Y_{j,k}\middle|N\right]\quad\text{(by Lemma 2)}\\
&=\frac{2}{N}\sum_{j=1}^{J}\sum_{k=1}^{K_j}\mathbb{E}\left[(W^1_{j,k}-W^0_{j,k})Y_{j,k}\middle|N\right]\\
&=\frac{2\mathcal{J}}{N}\mathbb{E}\left[(W^1_{j,k}-W^0_{j,k})Y_{j,k}\middle|N\right]\\
&=\frac{2\mathcal{J}}{N}\mathbb{E}\Big[\big(\mathcal{I}(A^m_{j,k}=1,Z^m_j=1)-\mathcal{I}(A^m_{j,k}=0,Z^m_j=1)\big)Y_{j,k}\Big|N\Big]\\
&\quad+\frac{2\mathcal{J}}{N}\mathbb{E}\Big[\big(\mathcal{I}(A^p_{j,k}=1,Z^p_j=1)-\mathcal{I}(A^p_{j,k}=0,Z^p_j=1)\big)Y_{j,k}\Big|N\Big]\\
&=\frac{2\mathcal{J}}{N}\mathbb{E}\left[\begin{pmatrix}\mathcal{I}(A^m_{j,k}=1,Z^m_j=1)\\-\mathcal{I}(A^m_{j,k}=0,Z^m_j=1)\end{pmatrix}\begin{pmatrix}Y^m_{j,k}(1)\mathcal{I}(A^m_{j,k}=1)\\+Y^m_{j,k}(0)\mathcal{I}(A^m_{j,k}=0)\end{pmatrix}\middle|N\right]\\
&\quad+\frac{2\mathcal{J}}{N}\mathbb{E}\left[\begin{pmatrix}\mathcal{I}(A^p_{j,k}=1,Z^p_j=1)\\-\mathcal{I}(A^p_{j,k}=0,Z^p_j=1)\end{pmatrix}\begin{pmatrix}Y^p_{j,k}(1)\mathcal{I}(A^p_{j,k}=1)\\+Y^p_{j,k}(0)\mathcal{I}(A^p_{j,k}=0)\end{pmatrix}\middle|N\right]\\
&=\frac{2\mathcal{J}}{N}\mathbb{E}\left[\mathcal{I}(A^m_{j,k}=1,Z^m_j=1)Y^m_{j,k}(1)-\mathcal{I}(A^m_{j,k}=0,Z^m_j=1)Y^m_{j,k}(0)\middle|N\right]
\end{aligned}$$

$$
\begin{aligned}
&+ \frac{2\mathcal{J}}{N}\mathbb{E}\left[\mathcal{I}(A^p_{j,k}=1, Z^p_j=1)Y^p_{j,k}(1) - \mathcal{I}(A^p_{j,k}=0, Z^p_j=1)Y^p_{j,k}(0)\middle| N\right] \\
&= \frac{2\mathcal{J}}{N}\mathbb{E}\Big[\big(\mathcal{I}(A^m_{j,k}=1)Y^m_{j,k}(1) - \mathcal{I}(A^m_{j,k}=0)Y^m_{j,k}(0)\big)\mathcal{I}(Z^m_j=1)\Big|N\Big] \\
&\quad + \frac{2\mathcal{J}}{N}\mathbb{E}\Big[\big(\mathcal{I}(A^p_{j,k}=1)Y^p_{j,k}(1) - \mathcal{I}(A^p_{j,k}=0)Y^p_{j,k}(0)\big)\mathcal{I}(Z^p_j=1)\Big|N\Big] \\
&= \frac{2\mathcal{J}}{N}\mathbb{P}(Z^m_j=1|N)\mathbb{E}\big[\mathcal{I}(A^m_{j,k}=1)Y^m_{j,k}(1) - \mathcal{I}(A^m_{j,k}=0)Y^m_{j,k}(0)\big|Z^m_j=1,N\big] \\
&+ \frac{2\mathcal{J}}{N}\mathbb{P}(Z^p_j=1|N)\mathbb{E}\big[\mathcal{I}(A^p_{j,k}=1)Y^p_{j,k}(1) - \mathcal{I}(A^p_{j,k}=0)Y^p_{j,k}(0)\big|Z^p_j=1,N\big] \qquad \text{(S9)} \\
&= \frac{2\mathcal{J}}{N}\frac{N}{2\mathcal{J}}\mathbb{E}\big[\mathcal{I}(A^m_{j,k}=1)Y^m_{j,k}(1) - \mathcal{I}(A^m_{j,k}=0)Y^m_{j,k}(0)\big|Z^m_j=1,N\big] \\
&\quad + \frac{2\mathcal{J}}{N}\frac{N}{2\mathcal{J}}\mathbb{E}\big[\mathcal{I}(A^p_{j,k}=1)Y^p_{j,k}(1) - \mathcal{I}(A^p_{j,k}=0)Y^p_{j,k}(0)\big|Z^p_j=1,N\big] \qquad \text{(S10)} \\
&= \mathbb{E}\big[\mathcal{I}(A^m_{j,k}=1)Y^m_{j,k}(1)\big|Z^m_j=1,N\big] - \mathbb{E}[\mathcal{I}(A^m_{j,k}=0)Y^m_{j,k}(0)\big|Z^m_j=1,N\big] \\
&\quad + \mathbb{E}\big[\mathcal{I}(A^p_{j,k}=1)Y^p_{j,k}(1)\big|Z^p_j=1,N\big] - \mathbb{E}[\mathcal{I}(A^p_{j,k}=0)Y^p_{j,k}(0)\big|Z^p_j=1,N\big] \\
&= \mathbb{E}\big[\mathcal{I}(A^m_{j,k}=1)\big|Z^m_j=1,N\big]\mathbb{E}\big[Y^m_{j,k}(1)\big|Z^m_j=1,N\big] \\
&\quad - \mathbb{E}\big[\mathcal{I}(A^m_{j,k}=0)\big|Z^m_j=1,N\big]\mathbb{E}\big[Y^m_{j,k}(0)\big|Z^m_j=1,N\big] \\
&\quad + \mathbb{E}\big[\mathcal{I}(A^p_{j,k}=1)\big|Z^p_j=1,N\big]\mathbb{E}\big[Y^p_{j,k}(1)\big|Z^p_j=1,N\big] \\
&\quad - \mathbb{E}\big[\mathcal{I}(A^p_{j,k}=0)\big|Z^p_j=1,N\big]\mathbb{E}\big[Y^p_{j,k}(0)\big|Z^p_j=1,N\big] \\
&= \mathbb{P}(A^m_{j,k}=1|Z^m_j=1,N)\mathbb{E}\big[Y^m_{j,k}(1)\big|Z^m_j=1,N\big] \\
&\quad - \mathbb{P}(A^m_{j,k}=0|Z^m_j=1,N)\mathbb{E}\big[Y^m_{j,k}(0)\big|Z^m_j=1,N\big] \\
&\quad + \mathbb{P}(A^p_{j,k}=1|Z^p_j=1,N)\mathbb{E}\big[Y^p_{j,k}(1)\big|Z^p_j=1,N\big] \\
&\quad - \mathbb{P}(A^p_{j,k}=0|Z^p_j=1,N)\mathbb{E}\big[Y^p_{j,k}(0)\big|Z^p_j=1,N\big] \\
&= \frac{1}{2}\mathbb{E}\big[Y^m_{j,k}(1)\big|Z^m_j=1,N\big] - \frac{1}{2}\mathbb{E}\big[Y^m_{j,k}(0)\big|Z^m_j=1,N\big] \\
&\quad + \frac{1}{2}\mathbb{E}\big[Y^p_{j,k}(1)\big|Z^p_j=1,N\big] - \frac{1}{2}\mathbb{E}\big[Y^p_{j,k}(0)\big|Z^p_j=1,N\big] \\
&= \frac{1}{2}\mathbb{E}[Y^m(1)-Y^m(0)|Z^m=1,N] + \frac{1}{2}\mathbb{E}[Y^p(1)-Y^p(0)|Z^m=1,N] \\
&= \delta_{\text{TMT}}
\end{aligned}
$$

Eq (S9) equals Eq (S10) because $\mathbb{P}(Z^m=1|N) = \mathbb{P}(Z^p=1|N) = N/(2\mathcal{J})$, which follows because there are N parent-offspring pairs with heterozygous parental genotype among $2\mathcal{J}$ parent-offspring pairs in the whole sample.

A.8 Proof of Theorem 3

Part A. We first discuss the covariance between siblings within the same family. Let $D_{j,k} = (W^1_{j,k} - W^0_{j,k})(Y_{j,k} - \mu)$ for offspring k in family j and $D_{j,h} = (W^1_{j,h} - W^0_{j,h})(Y_{j,h} - \mu)$ for offspring

h in family j. Here we derive a closed formula for $\mathbb{C}(D_{j,k}, D_{j,h}|N)$ where $k \neq h$. We show that when the null hypothesis is true $\mathbb{C}(D_{j,k}, D_{j,h}|N) = 0$ and in general $\mathbb{C}(D_{j,k}, D_{j,h}|N) \geq 0$. Let $W^0_{j,k} = W^{0m}_{j,k} + W^{0p}_{j,k}$, $W^1_{j,k} = W^{1m}_{j,k} + W^{1p}_{j,k}$ where

$$\begin{aligned} W^{0m}_{j,k} &= \mathcal{I}(A^m_{j,k} = 0, Z^m_j = 1), & W^{0p}_{j,k} &= \mathcal{I}(A^p_{j,k} = 0, Z^p_j = 1), \\ W^{1m}_{j,k} &= \mathcal{I}(A^m_{j,k} = 1, Z^m_j = 1), & W^{1p}_{j,k} &= \mathcal{I}(A^p_{j,k} = 1, Z^p_j = 1), \end{aligned}$$

and also $W^0_{j,h} = W^{0m}_{j,h} + W^{0p}_{j,h}$, $W^1_{j,h} = W^{1m}_{j,h} + W^{1p}_{j,h}$ where

$$\begin{aligned} W^{0m}_{j,h} &= \mathcal{I}(A^m_{j,h} = 0, Z^m_j = 1), & W^{0p}_{j,h} &= \mathcal{I}(A^p_{j,h} = 0, Z^p_j = 1), \\ W^{1m}_{j,h} &= \mathcal{I}(A^m_{j,h} = 1, Z^m_j = 1), & W^{1p}_{j,h} &= \mathcal{I}(A^p_{j,h} = 1, Z^p_j = 1). \end{aligned}$$

Then

$$\begin{aligned} \mathbb{C}(D_{j,k}, D_{j,h}|N) &= \mathbb{C}\left(\begin{array}{c} \big(W^{1m}_{j,k} + W^{1p}_{j,k} - W^{0m}_{j,k} - W^{0p}_{j,k}\big)\big(Y_{j,k} - \mu\big), \\ \big(W^{1m}_{j,h} + W^{1p}_{j,h} - W^{0m}_{j,h} - W^{0p}_{j,h}\big)\big(Y_{j,h} - \mu\big) \end{array} \middle| N \right) \qquad \text{(S11)} \\ &= \mathbb{C}\Big(\big(W^{1m}_{j,k} - W^{0m}_{j,k}\big)\big(Y_{j,k} - \mu\big), \big(W^{1m}_{j,h} - W^{0m}_{j,h}\big)\big(Y_{j,h} - \mu\big)\Big|N\Big) \\ &\quad + \mathbb{C}\Big(\big(W^{1p}_{j,k} - W^{0p}_{j,k}\big)\big(Y_{j,k} - \mu\big), \big(W^{1p}_{j,h} - W^{0p}_{j,h}\big)\big(Y_{j,h} - \mu\big)\Big|N\Big) \\ &\quad + \mathbb{C}\Big(\big(W^{1m}_{j,k} - W^{0m}_{j,k}\big)\big(Y_{j,k} - \mu\big), \big(W^{1p}_{j,h} - W^{0p}_{j,h}\big)\big(Y_{j,h} - \mu\big)\Big|N\Big) \\ &\quad + \mathbb{C}\Big(\big(W^{1p}_{j,k} - W^{0p}_{j,k}\big)\big(Y_{j,k} - \mu\big), \big(W^{1m}_{j,h} - W^{0m}_{j,h}\big)\big(Y_{j,h} - \mu\big)\Big|N\Big). \end{aligned}$$

Eq (S11) involves four covariances whose calculations follow the same algebra. For the first covariance,

$$\begin{aligned} &\mathbb{C}\Big(\big(W^{1m}_{j,k} - W^{0m}_{j,k}\big)\big(Y_{j,k} - \mu\big), \big(W^{1m}_{j,h} - W^{0m}_{j,h}\big)\big(Y_{j,h} - \mu\big)\Big|N\Big) \qquad \text{(S12)} \\ &= \mathbb{C}\left(\begin{bmatrix} \mathcal{I}(A^m_{j,k} = 1, Z^m_j = 1) \\ -\mathcal{I}(A^m_{j,k} = 0, Z^m_j = 1) \end{bmatrix} \begin{bmatrix} \big(Y^m_{j,k}(1) - \mu\big)\mathcal{I}(A^m_{j,k} = 1) \\ +\big(Y^m_{j,k}(0) - \mu\big)\mathcal{I}(A^m_{j,k} = 0) \end{bmatrix}, \right. \\ &\qquad \left. \begin{bmatrix} \mathcal{I}(A^m_{j,h} = 1, Z^m_j = 1) \\ -\mathcal{I}(A^m_{j,h} = 0, Z^m_j = 1) \end{bmatrix} \begin{bmatrix} \big(Y^m_{j,h}(1) - \mu\big)\mathcal{I}(A^m_{j,h} = 1) \\ +\big(Y^m_{j,h}(0) - \mu\big)\mathcal{I}(A^m_{j,h} = 0) \end{bmatrix} \middle| N \right) \\ &= \mathbb{C}\Big(\big(Y^m_{j,k}(1) - \mu\big)\mathcal{I}(A^m_{j,k} = 1, Z^m_j = 1) - \big(Y^m_{j,k}(0) - \mu\big)\mathcal{I}(A^m_{j,k} = 0, Z^m_j = 1), \\ &\qquad \big(Y^m_{j,h}(1) - \mu\big)\mathcal{I}(A^m_{j,h} = 1, Z^m_j = 1) - \big(Y^m_{j,h}(0) - \mu\big)\mathcal{I}(A^m_{j,h} = 0, Z^m_j = 1)\Big|N\Big) \\ &= \mathbb{C}\Big((Y^m_{j,k}(1) - \mu)\mathcal{I}(A^m_{j,k} = 1, Z^m_j = 1), (Y^m_{j,h}(1) - \mu)\mathcal{I}(A^m_{j,h} = 1, Z^m_j = 1)\Big|N\Big) \qquad \text{(S13)} \\ &\quad + \mathbb{C}\Big(- (Y^m_{j,k}(0) - \mu)\mathcal{I}(A^m_{j,k} = 0, Z^m_j = 1), -(Y^m_{j,h}(0) - \mu)\mathcal{I}(A^m_{j,h} = 0, Z^m_j = 1)\Big|N\Big) \qquad \text{(S14)} \\ &\quad + \mathbb{C}\Big((Y^m_{j,k}(1) - \mu)\mathcal{I}(A^m_{j,k} = 1, Z^m_j = 1), -(Y^m_{j,h}(0) - \mu)\mathcal{I}(A^m_{j,h} = 0, Z^m_j = 1)\Big|N\Big) \qquad \text{(S15)} \end{aligned}$$

$$+\mathbb{C}\Big(-(Y^m_{j,k}(0)-\mu)\mathcal{I}(A^m_{j,k}=0,Z^m_j=1),(Y^m_{j,h}(1)-\mu)\mathcal{I}(A^m_{j,h}=1,Z^m_j=1)\Big|N\Big). \tag{S16}$$

We first calculate Eq (S13) as follows.

$$\begin{aligned}
&\mathbb{C}\Big((Y^m_{j,k}(1)-\mu)\mathcal{I}(A^m_{j,k}=1,Z^m_j=1),(Y^m_{j,h}(1)-\mu)\mathcal{I}(A^m_{j,h}=1,Z^m_j=1)\Big|N\Big)\\
&=\mathbb{E}\Big[(Y^m_{j,k}(1)-\mu)\mathcal{I}(A^m_{j,k}=1,Z^m_j=1)(Y^m_{j,h}(1)-\mu)\mathcal{I}(A^m_{j,h}=1,Z^m_j=1)\Big|N\Big]\\
&\quad-\mathbb{E}\Big[(Y^m_{j,k}(1)-\mu)\mathcal{I}(A^m_{j,k}=1,Z^m_j=1)\Big|N\Big]\mathbb{E}\Big[(Y^m_{j,h}(1)-\mu)\mathcal{I}(A^m_{j,h}=1,Z^m_j=1)\Big|N\Big]\\
&=\mathbb{E}\Big[(Y^m_{j,k}(1)-\mu)(Y^m_{j,h}(1)-\mu)\Big|A^m_{j,k}=A^m_{j,h}=1,Z^m_j=1,N\Big]\mathbb{P}\Big(A^m_{j,k}=A^m_{j,h}=1,Z^m_j=1\Big|N\Big)\\
&\quad-\mathbb{E}\Big[Y^m_{j,k}(1)-\mu\Big|A^m_{j,k}=1,Z^m_j=1,N\Big]\mathbb{P}\Big(A^m_{j,k}=1,Z^m_j=1\Big|N\Big) \qquad \text{(S17)}\\
&\quad\times\mathbb{E}\Big[Y^m_{j,h}(1)-\mu\Big|A^m_{j,h}=1,Z^m_j=1,N\Big]\mathbb{P}\Big(A^m_{j,h}=1,Z^m_j=1\Big|N\Big)\\
&=\mathbb{E}\Big[(Y^m_{j,k}(1)-\mu)(Y^m_{j,h}(1)-\mu)\Big|A^m_{j,k}=A^m_{j,h}=1,Z^m_j=1,N\Big]\frac{1}{4}\mathbb{P}(Z^m_j=1|N)\\
&\quad-\mathbb{E}\Big[Y^m_{j,k}(1)-\mu\Big|A^m_{j,k}=1,Z^m_j=1,N\Big]\frac{1}{2}\mathbb{P}(Z^m_j=1|N) \qquad \text{(S18)}\\
&\quad\times\mathbb{E}\Big[Y^m_{j,h}(1)-\mu\Big|A^m_{j,h}=1,Z^m_j=1,N\Big]\frac{1}{2}\mathbb{P}(Z^m_j=1|N)\\
&=\frac{1}{4}\mathbb{E}\Big[(Y^m_{j,k}(1)-\mu)(Y^m_{j,h}(1)-\mu)\Big|Z^m_j=1,N\Big]\mathbb{P}(Z^m_j=1|N)\\
&\quad-\frac{1}{2}\mathbb{E}\Big[Y^m_{j,k}(1)-\mu\Big|Z^m_j=1,N\Big]\mathbb{P}(Z^m_j=1|N) \qquad \text{(S19)}\\
&\quad\times\frac{1}{2}\mathbb{E}\Big[Y^m_{j,h}(1)-\mu\Big|Z^m_j=1,N\Big]\mathbb{P}(Z^m_j=1|N)\\
&=\frac{1}{4}\mathbb{E}\Big[(Y^m_{j,k}(1)-\mu)\mathcal{I}(Z^m_j=1)(Y^m_{j,h}(1)-\mu)\mathcal{I}(Z^m_j=1)\Big|N\Big]\\
&\quad-\frac{1}{4}\mathbb{E}\Big[(Y^m_{j,k}(1)-\mu)\mathcal{I}(Z^m_j=1)\Big|N\Big]\mathbb{E}\Big[(Y^m_{j,h}(1)-\mu)\mathcal{I}(Z^m_j=1)\Big|N\Big]\\
&=\frac{1}{4}\mathbb{C}\Big((Y^m_{j,k}(1)-\mu)\mathcal{I}(Z^m_j=1),(Y^m_{j,h}(1)-\mu)\mathcal{I}(Z^m_j=1)\Big|N\Big)
\end{aligned}$$

Eq (S17) to Eq (S18) follows from

$$\begin{aligned}
\mathbb{P}(A^m_{j,k}=A^m_{j,h}=1,Z^m_j=1|N)&=\mathbb{P}(A^m_{j,k}=A^m_{j,h}=1|Z^m_j=1,N)\mathbb{P}(Z^m_j=1|N)=\frac{1}{4}\mathbb{P}(Z^m_j=1|N),\\
\mathbb{P}(A^m_{j,k}=1,Z^m_j=1|N)&=\mathbb{P}(A^m_{j,k}=1|Z^m_j=1,N)\mathbb{P}(Z^m_j=1|N)=\frac{1}{2}\mathbb{P}(Z^m_j=1|N),\\
\mathbb{P}(A^m_{j,h}=1,Z^m_j=1|N)&=\mathbb{P}(A^m_{j,h}=1|Z^m_j=1,N)\mathbb{P}(Z^m_j=1|N)=\frac{1}{2}\mathbb{P}(Z^m_j=1|N).
\end{aligned}$$

Eq (S18) to Eq (S19) follows from Lemma 1 that $Y^m_{j,k}(1)\perp\!\!\!\perp A^m_{j,k}|Z^m_j=1$ and $Y^m_{j,h}(1)\perp\!\!\!\perp A^m_{j,h}|Z^m_j=1$. We use the same approach to calculate the other three covariances in Eq (S12) as follows.

$$\text{Eq (S14)}=\frac{1}{4}\mathbb{C}\Big(-(Y^m_{j,k}(0)-\mu)\mathcal{I}(Z^m_j=1),-(Y^m_{j,h}(0)-\mu)\mathcal{I}(Z^m_j=1)\Big|N\Big)$$

$$\text{Eq (S15)} = \frac{1}{4}\mathbb{C}\Big((Y^m_{j,k}(1)-\mu)\mathcal{I}(Z^m_j=1), -(Y^m_{j,h}(0)-\mu)\mathcal{I}(Z^m_j=1)\Big|N\Big)$$
$$\text{Eq (S16)} = \frac{1}{4}\mathbb{C}\Big(-(Y^m_{j,k}(0)-\mu)\mathcal{I}(Z^m_j=1), (Y^m_{j,h}(1)-\mu)\mathcal{I}(Z^m_j=1)\Big|N\Big)$$

We plug these four covariances into Eq (S12) to derive the following covariance.

$$\begin{aligned}
&\mathbb{C}\Big(\big(W^{1m}_{j,k}-W^{0m}_{j,k}\big)\big(Y_{j,k}-\mu\big), \big(W^{1m}_{j,h}-W^{0m}_{j,h}\big)\big(Y_{j,h}-\mu\big)\Big|N\Big)\\
&= \text{Eq (S13)} + \text{Eq (S14)} + \text{Eq (S15)} + \text{Eq (S16)}\\
&= \frac{1}{4}\mathbb{C}\Big(\begin{matrix}(Y^m_{j,k}(1)-\mu)\mathcal{I}(Z^m_j=1)-(Y^m_{j,k}(0)-\mu)\mathcal{I}(Z^m_j=1),\\ (Y^m_{j,h}(1)-\mu)\mathcal{I}(Z^m_j=1)-(Y^m_{j,h}(0)-\mu)\mathcal{I}(Z^m_j=1)\end{matrix}\Big|N\Big)\\
&= \frac{1}{4}\mathbb{C}\Big(\big(Y^m_{j,k}(1)-Y^m_{j,k}(0)\big)\mathcal{I}(Z^m_j=1), \big(Y^m_{j,h}(1)-Y^m_{j,h}(0)\big)\mathcal{I}(Z^m_j=1)\Big|N\Big)\\
&= \frac{1}{4}\mathbb{E}\Big[\mathbb{C}\big(Y^m_{j,k}(1)-Y^m_{j,k}(0), Y^m_{j,h}(1)-Y^m_{j,h}(0)\big|Z^m_j=1,N\big)\Big|N\Big]\\
&\quad+\frac{1}{4}\mathbb{C}\left(\begin{matrix}\mathbb{E}[Y^m_{j,k}(1)-Y^m_{j,k}(0)|Z^m_j=1,N],\\ \mathbb{E}[Y^m_{j,h}(1)-Y^m_{j,h}(0)|Z^m_j=1,N]\end{matrix}\middle|N\right) \qquad \text{(by the Law of Total Covariance)}\\
&= \frac{1}{4}\mathbb{E}\left[\mathbb{C}\left(\begin{matrix}(\alpha_2-2\alpha_1+\alpha_0)A^p_{j,k}+(\alpha_1-\alpha_0),\\ (\alpha_2-2\alpha_1+\alpha_0)A^p_{j,h}+(\alpha_1-\alpha_0)\end{matrix}\middle|Z^m_j=1,N\right)\middle|N\right]\\
&\quad+\frac{1}{4}\mathbb{C}\left(\begin{matrix}\mathbb{E}[(\alpha_2-2\alpha_1+\alpha_0)A^p_{j,k}+(\alpha_1-\alpha_0)|Z^m_j=1,N],\\ \mathbb{E}[(\alpha_2-2\alpha_1+\alpha_0)A^p_{j,h}+(\alpha_1-\alpha_0)|Z^m_j=1,N]\end{matrix}\middle|N\right) \qquad \text{(by Eq (11))}\\
&= \frac{(\alpha_2-2\alpha_1+\alpha_0)^2}{4}\mathbb{E}[\mathbb{C}(A^p_{j,k},A^p_{j,h}|Z^m_j=1,N)|\,N]\\
&\quad+\frac{(\alpha_2-2\alpha_1+\alpha_0)^2}{4}\mathbb{C}\left(\mathbb{E}[A^p_{j,k}|Z^m_j=1,N],\mathbb{E}[A^p_{j,h}|Z^m_j=1,N]|\,N\right)\\
&= \frac{(\alpha_2-2\alpha_1+\alpha_0)^2}{4}\mathbb{C}(A^p_{j,k},A^p_{j,h}|N) \qquad \text{(by the Law of Total Covariance)}
\end{aligned}$$

We use the same approach to calculate the other three covariances in Eq (S11) and add these four covariances to derive $\mathbb{C}(D_{j,k},D_{j,h}|N)$ as follows.

$$\begin{aligned}
\mathbb{C}(D_{j,k},D_{j,h}|N) &= \frac{(\alpha_2-2\alpha_1+\alpha_0)^2}{4}\mathbb{C}(A^p_{j,k},A^p_{j,h}|N)+\frac{(\alpha_2-2\alpha_1+\alpha_0)^2}{4}\mathbb{C}(A^m_{j,k},A^m_{j,h}|N)\\
&\quad+\frac{(\alpha_2-2\alpha_1+\alpha_0)^2}{4}\mathbb{C}(A^p_{j,k},A^m_{j,h}|N)+\frac{(\alpha_2-2\alpha_1+\alpha_0)^2}{4}\mathbb{C}(A^m_{j,k},A^p_{j,h}|N)\\
&= \frac{(\alpha_2-2\alpha_1+\alpha_0)^2}{4}\left(\begin{matrix}\mathbb{C}(A^p_{j,k},A^p_{j,h}|N)+\mathbb{C}(A^m_{j,k},A^m_{j,h}|N)\\ +\mathbb{C}(A^p_{j,k},A^m_{j,h}|N)+\mathbb{C}(A^m_{j,k},A^p_{j,h}|N)\end{matrix}\right)\\
&= \frac{(\alpha_2-2\alpha_1+\alpha_0)^2}{4}\mathbb{C}(A^p_{j,k}+A^m_{j,k},A^p_{j,h}+A^m_{j,h}|N)\\
&= \frac{(\alpha_2-2\alpha_1+\alpha_0)^2}{4}\mathbb{C}(G_{j,k},G_{j,h}|N)
\end{aligned}$$

When the null hypothesis of no causality is true, $\alpha_0 = \alpha_1 = \alpha_2$. Therefore, $(\alpha_2 - 2\alpha_1 + \alpha_0) = 0$ and $\mathbb{C}(D_{j,k}, D_{j,h}|N) = 0$ when the null hypothesis of no causality is true. In general, $(\alpha_2 - 2\alpha_1 + \alpha_0)^2 \geq 0$. The property that $\mathbb{C}(G_{j,k}, G_{j,h}|N) \geq 0$ is satisfied in prevalent population genetics models including the Identical-By-Descent (IBD) model, the co-ancestry model, etc [1]. In this case, $\mathbb{C}(D_{j,k}, D_{j,h}|N) \geq 0$ in general.

Part B. We now consider the covariance between offspring from different families. Let $D_{j,k} = (W^1_{j,k} - W^0_{j,k})(Y_{j,k} - \mu)$ for offspring k in family j and $D_{l,s} = (W^1_{l,s} - W^0_{l,s})(Y_{l,s} - \mu)$ for offspring s in family l. Here we derive a closed formula for $\mathbb{C}(D_{j,k}, D_{l,s}|N)$ where $j \neq l$. We show that when the null hypothesis is true, $\mathbb{C}(D_{j,k}, D_{l,s}|N) = 0$ and in general $\mathbb{C}(D_{j,k}, D_{l,s}|N) \geq 0$. For offspring k in family j, let $W^0_{j,k} = W^{0m}_{j,k} + W^{0p}_{j,k}$, $W^1_{j,k} = W^{1m}_{j,k} + W^{1p}_{j,k}$ where

$$\begin{aligned}
W^{0m}_{j,k} &= \mathcal{I}(A^m_{j,k} = 0, Z^m_j = 1), & W^{0p}_{j,k} &= \mathcal{I}(A^p_{j,k} = 0, Z^p_j = 1),\\
W^{1m}_{j,k} &= \mathcal{I}(A^m_{j,k} = 1, Z^m_j = 1), & W^{1p}_{j,k} &= \mathcal{I}(A^p_{j,k} = 1, Z^p_j = 1).
\end{aligned}$$

For offspring s in family l, let $W^0_{l,s} = W^{0m}_{l,s} + W^{0p}_{l,s}$, $W^1_{l,s} = W^{1m}_{l,s} + W^{1p}_{l,s}$ where

$$\begin{aligned}
W^{0m}_{l,s} &= \mathcal{I}(A^m_{l,s} = 0, Z^m_l = 1), & W^{0p}_{l,s} &= \mathcal{I}(A^p_{l,s} = 0, Z^p_l = 1),\\
W^{1m}_{l,s} &= \mathcal{I}(A^m_{l,s} = 1, Z^m_l = 1), & W^{1p}_{l,s} &= \mathcal{I}(A^p_{l,s} = 1, Z^p_l = 1).
\end{aligned}$$

Then

$$\begin{aligned}
\mathbb{C}(D_{j,k}, D_{l,s}|N) &= \mathbb{C}\Big(\begin{array}{l} \big(W^{1m}_{j,k} + W^{1p}_{j,k} - W^{0m}_{j,k} - W^{0p}_{j,k}\big)\big(Y_{j,k} - \mu\big), \\ \big(W^{1m}_{l,s} + W^{1p}_{l,s} - W^{0m}_{l,s} - W^{0p}_{l,s}\big)\big(Y_{l,s} - \mu\big) \end{array} \Big| N\Big) \qquad \text{(S20)}\\
&= \mathbb{C}\Big(\big(W^{1m}_{j,k} - W^{0m}_{j,k}\big)\big(Y_{j,k} - \mu\big), \big(W^{1m}_{l,s} - W^{0m}_{l,s}\big)\big(Y_{l,s} - \mu\big)\Big| N\Big)\\
&\quad + \mathbb{C}\Big(\big(W^{1p}_{j,k} - W^{0p}_{j,k}\big)\big(Y_{j,k} - \mu\big), \big(W^{1p}_{l,s} - W^{0p}_{l,s}\big)\big(Y_{l,s} - \mu\big)\Big| N\Big)\\
&\quad + \mathbb{C}\Big(\big(W^{1m}_{j,k} - W^{0m}_{j,k}\big)\big(Y_{j,k} - \mu\big), \big(W^{1p}_{l,s} - W^{0p}_{l,s}\big)\big(Y_{l,s} - \mu\big)\Big| N\Big)\\
&\quad + \mathbb{C}\Big(\big(W^{1p}_{j,k} - W^{0p}_{j,k}\big)\big(Y_{j,k} - \mu\big), \big(W^{1m}_{l,s} - W^{0m}_{l,s}\big)\big(Y_{l,s} - \mu\big)\Big| N\Big).
\end{aligned}$$

Eq (S20) involves four covariances whose calculations follow the same algebra. We calculate the first covariance as follows.

$$\begin{aligned}
&\mathbb{C}\Big(\big(W^{1m}_{j,k} - W^{0m}_{j,k}\big)\big(Y_{j,k} - \mu\big), \big(W^{1m}_{l,s} - W^{0m}_{l,s}\big)\big(Y_{l,s} - \mu\big)\Big| N\Big) \qquad \text{(S21)}\\
&= \mathbb{C}\Bigg(\left[\begin{array}{l} \mathcal{I}(A^m_{j,k} = 1, Z^m_j = 1) \\ \quad -\mathcal{I}(A^m_{j,k} = 0, Z^m_j = 1) \end{array}\right]\left[\begin{array}{l} \big(Y^m_{j,k}(1) - \mu\big)\mathcal{I}(A^m_{j,k} = 1) \\ \quad +\big(Y^m_{j,k}(0) - \mu\big)\mathcal{I}(A^m_{j,k} = 0) \end{array}\right],
\end{aligned}$$

$$\left[\begin{array}{l}\mathcal{I}(A^m_{l,s}=1, Z^m_l=1)\\ \quad -\mathcal{I}(A^m_{l,s}=0, Z^m_l=1)\end{array}\right]\left[\begin{array}{l}\big(Y^m_{l,s}(1)-\mu\big)\mathcal{I}(A^m_{l,s}=1)\\ \quad +\big(Y^m_{l,s}(0)-\mu\big)\mathcal{I}(A^m_{l,s}=0)\end{array}\right]\Bigg|N\Bigg)$$

$$
\begin{aligned}
&= \mathbb{C}\Big(\big(Y^m_{j,k}(1)-\mu\big)\mathcal{I}(A^m_{j,k}=1, Z^m_j=1) - \big(Y^m_{j,k}(0)-\mu\big)\mathcal{I}(A^m_{j,k}=0, Z^m_j=1),\\
&\qquad \big(Y^m_{l,s}(1)-\mu\big)\mathcal{I}(A^m_{l,s}=1, Z^m_l=1) - \big(Y^m_{l,s}(0)-\mu\big)\mathcal{I}(A^m_{l,s}=0, Z^m_l=1)\Big|N\Big)\\
&= \mathbb{C}\Big((Y^m_{j,k}(1)-\mu)\mathcal{I}(A^m_{j,k}=1, Z^m_j=1), (Y^m_{l,s}(1)-\mu)\mathcal{I}(A^m_{l,s}=1, Z^m_l=1)\Big|N\Big) \qquad \text{(S22)}\\
&\quad + \mathbb{C}\Big(-(Y^m_{j,k}(0)-\mu)\mathcal{I}(A^m_{j,k}=0, Z^m_j=1), -(Y^m_{l,s}(0)-\mu)\mathcal{I}(A^m_{l,s}=0, Z^m_l=1)\Big|N\Big) \qquad \text{(S23)}\\
&\quad + \mathbb{C}\Big((Y^m_{j,k}(1)-\mu)\mathcal{I}(A^m_{j,k}=1, Z^m_j=1), -(Y^m_{l,s}(0)-\mu)\mathcal{I}(A^m_{l,s}=0, Z^m_l=1)\Big|N\Big) \qquad \text{(S24)}\\
&\quad + \mathbb{C}\Big(-(Y^m_{j,k}(0)-\mu)\mathcal{I}(A^m_{j,k}=0, Z^m_j=1), (Y^m_{l,s}(1)-\mu)\mathcal{I}(A^m_{l,s}=1, Z^m_l=1)\Big|N\Big) \qquad \text{(S25)}
\end{aligned}
$$

We first calculate Eq (S22) as follows.

$$
\begin{aligned}
&\mathbb{C}\Big((Y^m_{j,k}(1)-\mu)\mathcal{I}(A^m_{j,k}=1, Z^m_j=1), (Y^m_{l,s}(1)-\mu)\mathcal{I}(A^m_{l,s}=1, Z^m_l=1)\Big|N\Big)\\
&= \mathbb{E}\Big[(Y^m_{j,k}(1)-\mu)\mathcal{I}(A^m_{j,k}=1, Z^m_j=1)(Y^m_{l,s}(1)-\mu)\mathcal{I}(A^m_{l,s}=1, Z^m_l=1)\Big|N\Big]\\
&\quad - \mathbb{E}\Big[(Y^m_{j,k}(1)-\mu)\mathcal{I}(A^m_{j,k}=1, Z^m_j=1)\Big|N\Big]\mathbb{E}\Big[(Y^m_{l,s}(1)-\mu)\mathcal{I}(A^m_{l,s}=1, Z^m_l=1)\Big|N\Big]\\
&= \mathbb{E}\left[(Y^m_{j,k}(1)-\mu)(Y^m_{l,s}(1)-\mu)\left|\begin{array}{l}A^m_{j,k}=A^m_{l,s}=1,\\ Z^m_j=Z^m_l=1, N\end{array}\right.\right]\mathbb{P}\left(\begin{array}{l}A^m_{j,k}=A^m_{j,h}=1,\\ Z^m_j=Z^m_l=1\end{array}\middle| N\right)\\
&\quad - \mathbb{E}\Big[Y^m_{j,k}(1)-\mu\Big|A^m_{j,k}=1, Z^m_j=1, N\Big]\mathbb{P}(A^m_j=1, Z^m_j=1|N) \qquad \text{(S26)}\\
&\qquad \times \mathbb{E}\Big[Y^m_{l,s}(1)-\mu\Big|A^m_{l,s}=1, Z^m_j=1, N\Big]\mathbb{P}(A^m_l=1, Z^m_l=1|N)\\
&= \mathbb{E}\Big[(Y^m_{j,k}(1)-\mu)(Y^m_{l,s}(1)-\mu)\Big|A^m_{j,k}=A^m_{l,s}=1, Z^m_j=Z^m_l=1, N\Big]\frac{1}{4}\mathbb{P}(Z^m_j=Z^m_l=1|N)\\
&\quad - \mathbb{E}\Big[Y^m_{j,k}(1)-\mu\Big|A^m_{j,k}=1, Z^m_j=1, N\Big]\frac{1}{2}\mathbb{P}(Z^m_j=1|N) \qquad \text{(S27)}\\
&\qquad \times \mathbb{E}\Big[Y^m_{l,s}(1)-\mu\Big|A^m_{l,s}=1, Z^m_j=1, N\Big]\frac{1}{2}\mathbb{P}(Z^m_l=1|N)\\
&= \frac{1}{4}\mathbb{E}\Big[(Y^m_{j,k}(1)-\mu)(Y^m_{l,s}(1)-\mu)\Big|Z^m_j=Z^m_l=1, N\Big]\mathbb{P}(Z^m_j=Z^m_l=1|N)\\
&\quad - \frac{1}{2}\mathbb{E}\Big[Y^m_{j,k}(1)-\mu\Big|Z^m_j=1, N\Big]\mathbb{P}(Z^m_j=1|N) \qquad \text{(S28)}\\
&\qquad \times \frac{1}{2}\mathbb{E}\Big[Y^m_{l,s}(1)-\mu\Big|Z^m_l=1, N\Big]\mathbb{P}(Z^m_l=1|N)\\
&= \frac{1}{4}\mathbb{E}\Big[(Y^m_{j,k}(1)-\mu)\mathcal{I}(Z^m_j=1)(Y^m_{l,s}(1)-\mu)\mathcal{I}(Z^m_l=1)\Big|N\Big]\\
&\quad - \frac{1}{4}\mathbb{E}\Big[(Y^m_{j,k}(1)-\mu)\mathcal{I}(Z^m_j=1)\Big|N\Big]\mathbb{E}\Big[(Y^m_{l,s}(1)-\mu)\mathcal{I}(Z^m_l=1)\Big|N\Big]\\
&= \frac{1}{4}\mathbb{C}\Big((Y^m_{j,k}(1)-\mu)\mathcal{I}(Z^m_j=1), (Y^m_{l,s}(1)-\mu)\mathcal{I}(Z^m_l=1)\Big|N\Big)
\end{aligned}
$$

From Eq (S26) to Eq (S27) follows that

$$\mathbb{P}\left(\begin{array}{c} A^m_{j,k}=A^m_{l,s}=1 \\ Z^m_j=Z^m_l=1 \end{array}\middle| N\right)=\underbrace{\mathbb{P}\left(\begin{array}{c} A^m_{j,k}=1 \\ A^m_{l,s}=1 \end{array}\middle|\begin{array}{c} Z^m_j=1 \\ Z^m_l=1, N \end{array}\right)}_{=1/4}\mathbb{P}\left(\begin{array}{c} Z^m_j=1 \\ Z^m_l=1 \end{array}\middle| N\right),$$

$$\mathbb{P}(A^m_{j,k}=1, Z^m_j=1|N)=\underbrace{\mathbb{P}(A^m_{j,k}=1|Z^m_j=1,N)}_{=1/2}\mathbb{P}(Z^m_j=1|N),$$

$$\mathbb{P}(A^m_{l,s}=1, Z^m_l=1|N)=\underbrace{\mathbb{P}(A^m_{l,s}=1|Z^m_l=1,N)}_{=1/2}\mathbb{P}(Z^m_l=1|N).$$

Eq (S27) to Eq (S28) follows from Lemma 1 that $Y^m_{j,k}(1) \perp\!\!\!\perp A^m_{j,k}|Z^m_j=1$ and $Y^m_{l,s}(1) \perp\!\!\!\perp A^m_{l,s}|Z^m_l=1$. We use the same approach to calculate the other three covariances in Eq (S12) as follows.

$$\begin{aligned}
\text{Eq (S23)} &= \frac{1}{4}\mathbb{C}\Big(-(Y^m_{j,k}(0)-\mu)\mathcal{I}(Z^m_j=1), -(Y^m_{l,s}(0)-\mu)\mathcal{I}(Z^m_l=1)\Big|N\Big)\\
\text{Eq (S24)} &= \frac{1}{4}\mathbb{C}\Big((Y^m_{j,k}(1)-\mu)\mathcal{I}(Z^m_j=1), -(Y^m_{l,s}(0)-\mu)\mathcal{I}(Z^m_l=1)\Big|N\Big)\\
\text{Eq (S25)} &= \frac{1}{4}\mathbb{C}\Big(-(Y^m_{j,k}(0)-\mu)\mathcal{I}(Z^m_j=1), (Y^m_{l,s}(1)-\mu)\mathcal{I}(Z^m_l=1)\Big|N\Big)
\end{aligned}$$

We plug these four covariances into Eq (S21) to derive the following.

$$\begin{aligned}
&\mathbb{C}\Big(\big(W^{1m}_{j,k}-W^{0m}_{j,k}\big)\big(Y_{j,k}-\mu\big), \big(W^{1m}_{l,s}-W^{0m}_{l,s}\big)\big(Y_{l,s}-\mu\big)\Big|N\Big)\\
&= \text{Eq (S22)} + \text{Eq (S23)} + \text{Eq (S24)} + \text{Eq (S25)}\\
&= \frac{1}{4}\mathbb{C}\left(\begin{array}{c} (Y^m_{j,k}(1)-\mu)\mathcal{I}(Z^m_j=1)-(Y^m_{j,k}(0)-\mu)\mathcal{I}(Z^m_j=1), \\ (Y^m_{l,s}(1)-\mu)\mathcal{I}(Z^m_l=1)-(Y^m_{l,s}(0)-\mu)\mathcal{I}(Z^m_l=1) \end{array}\middle| N\right)\\
&= \frac{1}{4}\mathbb{C}\Big(\big(Y^m_{j,k}(1)-Y^m_{j,k}(0)\big)\mathcal{I}(Z^m_j=1), \big(Y^m_{l,s}(1)-Y^m_{l,s}(0)\big)\mathcal{I}(Z^m_l=1)\Big|N\Big)\\
&= \frac{1}{4}\mathbb{E}\Big[\mathbb{C}\big(Y^m_{j,k}(1)-Y^m_{j,k}(0), Y^m_{l,s}(1)-Y^m_{l,s}(0)\big|Z^m_j=Z^m_l=1,N\big)\Big|N\Big]\\
&\quad+\frac{1}{4}\mathbb{C}\left(\begin{array}{c} \mathbb{E}[Y^m_{j,k}(1)-Y^m_{j,k}(0)|Z^m_j=Z^m_l=1,N], \\ \mathbb{E}[Y^m_{l,s}(1)-Y^m_{l,s}(0)|Z^m_j=Z^m_l=1,N] \end{array}\middle| N\right) \text{ (by the Law of Total Covariance)}\\
&= \frac{1}{4}\mathbb{E}\left[\mathbb{C}\left(\begin{array}{c} (\alpha_2-2\alpha_1+\alpha_0)A^p_{j,k}+(\alpha_1-\alpha_0), \\ (\alpha_2-2\alpha_1+\alpha_0)A^p_{l,s}+(\alpha_1-\alpha_0) \end{array}\middle| Z^m_j=Z^m_l=1,N\right)\middle| N\right]\\
&\quad+\frac{1}{4}\mathbb{C}\left(\begin{array}{c} \mathbb{E}[(\alpha_2-2\alpha_1+\alpha_0)A^p_{j,k}+(\alpha_1-\alpha_0)|Z^m_j=Z^m_l=1,N], \\ \mathbb{E}[(\alpha_2-2\alpha_1+\alpha_0)A^p_{l,s}+(\alpha_1-\alpha_0)|Z^m_j=Z^m_l=1,N] \end{array}\middle| N\right) \quad \text{(by Eq (11))}\\
&= \frac{(\alpha_2-2\alpha_1+\alpha_0)^2}{4}\mathbb{E}[\mathbb{C}(A^p_{j,k}, A^p_{l,s}|Z^m_j=Z^m_l=1,N)|\,N]\\
&\quad+\frac{(\alpha_2-2\alpha_1+\alpha_0)^2}{4}\mathbb{C}\left(\mathbb{E}[A^p_{j,k}|Z^m_j=Z^m_l=1,N], \mathbb{E}[A^p_{l,s}|Z^m_j=Z^m_l=1,N]\middle| N\right)
\end{aligned}$$

$$= \frac{(\alpha_2 - 2\alpha_1 + \alpha_0)^2}{4} \mathbb{C}(A^p_{j,k}, A^p_{l,s}|N) \qquad \text{(by the Law of Total Covariance)}$$

We use the same approach to calculate the other three covariances in Eq (S20) and add these four covariances to derive $\mathbb{C}(D_{j,k}, D_{l,s}|N)$ as follows.

$$\begin{aligned}
\mathbb{C}(D_{j,k}, D_{l,s}|N) &= \frac{(\alpha_2 - 2\alpha_1 + \alpha_0)^2}{4} \mathbb{C}(A^p_{j,k}, A^p_{l,s}|N) + \frac{(\alpha_2 - 2\alpha_1 + \alpha_0)^2}{4} \mathbb{C}(A^m_{j,k}, A^m_{l,s}|N) \\
&\quad + \frac{(\alpha_2 - 2\alpha_1 + \alpha_0)^2}{4} \mathbb{C}(A^p_{j,k}, A^m_{l,s}|N) + \frac{(\alpha_2 - 2\alpha_1 + \alpha_0)^2}{4} \mathbb{C}(A^m_{j,k}, A^p_{l,s}|N) \\
&= \frac{(\alpha_2 - 2\alpha_1 + \alpha_0)^2}{4} \begin{pmatrix} \mathbb{C}(A^p_{j,k}, A^p_{l,s}|N) + \mathbb{C}(A^m_{j,k}, A^m_{l,s}|N) \\ +\mathbb{C}(A^p_{j,k}, A^m_{l,s}|N) + \mathbb{C}(A^m_{j,k}, A^p_{l,s}|N) \end{pmatrix} \\
&= \frac{(\alpha_2 - 2\alpha_1 + \alpha_0)^2}{4} \mathbb{C}(A^p_{j,k} + A^m_{j,k}, A^p_{l,s} + A^m_{l,s}|N) \\
&= \frac{(\alpha_2 - 2\alpha_1 + \alpha_0)^2}{4} \mathbb{C}(G_{j,k}, G_{l,s}|N)
\end{aligned}$$

When the null hypothesis of no causality is true, $\alpha_0 = \alpha_1 = \alpha_2$. Therefore, $(\alpha_2 - 2\alpha_1 + \alpha_0) = 0$ and $\mathbb{C}(D_{j,k}, D_{l,s}|N) = 0$ when the null hypothesis of no causality is true. In general, $(\alpha_2 - 2\alpha_1 + \alpha_0)^2 \geq 0$. Besides, $\mathbb{C}(G_{j,k}, G_{l,s}|N) \geq 0$ is assumed to be satisfied in prevalent population genetics models including the IBD model, the co-ancestry model, etc. Then $\mathbb{C}(D_{j,k}, D_{l,s}|N) \geq 0$ in general.

Part C. We derive a closed formula for $\mathbb{V}(d^{\text{pc}}_{\text{gTMT}}|N)$ and use Part A and B to complete the proof of Theorem 3. Let $\mathcal{J}$ be the total number of offspring in that $\mathcal{J} = \sum_{j=1}^{J} K_j$. Recall that $D_{j,k} = (W^1_{j,k} - W^0_{j,k})(Y_{j,k} - \mu)$ for offspring k in family j. Then $\mathbb{V}(d^{\text{pc}}_{\text{gTMT}}|N)$ equals

$$\begin{aligned}
&\mathbb{V}\left(\frac{2}{N} \sum_{j=1}^{J} \sum_{k=1}^{K_j} D_{j,k} \middle| N \right) \\
&= \frac{4}{N^2} \mathbb{V}\left(\sum_{j=1}^{J} \sum_{k=1}^{K_j} D_{j,k} \middle| N \right) \\
&= \frac{4}{N^2} \sum_{j=1}^{J} \mathbb{V}\left(\sum_{k=1}^{K_j} D_{j,k} \middle| N \right) + \frac{4}{N^2} \sum_{j,l,j \neq l} \mathbb{C}\left(\sum_{k=1}^{K_j} D_{j,k}, \sum_{s=1}^{K_l} D_{l,s} \middle| N \right) \qquad \text{(S29)} \\
&= \frac{4}{N^2} \sum_{j=1}^{J} \left(\sum_{k=1}^{K_j} \mathbb{V}\big(D_{j,k}|N\big) + \sum_{k,h,k \neq h} \mathbb{C}\big(D_{j,k}, D_{j,h}|N\big) \right) + \frac{4}{N^2} \sum_{j,l,j \neq l} \sum_{k=1}^{K_j} \sum_{s=1}^{K_l} \mathbb{C}\big(D_{j,k}, D_{l,s}|N\big) \\
&= \frac{4}{N^2} \sum_{j=1}^{J} \sum_{k=1}^{K_j} \mathbb{V}\big(D_{j,k}|N\big)
\end{aligned}$$

$$+\frac{4}{N^2}\left(\sum_{j=1}^{J}\sum_{k,h,k\neq h}\mathbb{C}\big(D_{j,k},D_{j,h}\big|N\big)+\sum_{j,l,j\neq l}\sum_{k=1}^{K_j}\sum_{s=1}^{K_l}\mathbb{C}\big(D_{j,k},D_{l,s}\big|N\big)\right)$$

$$=\frac{4}{N^2}\sum_{j=1}^{J}\sum_{k=1}^{K_j}\mathbb{E}\Big[\mathbb{V}\big(D_{j,k}\big|\mathcal{T}_0,\mathcal{T}_1,\mathcal{T}_{00},\mathcal{T}_{11},N\big)\Big|N\Big]$$

$$+\frac{4}{N^2}\sum_{j=1}^{J}\sum_{k=1}^{K_j}\mathbb{V}\Big(\mathbb{E}\big[D_{j,k}\big|\mathcal{T}_0,\mathcal{T}_1,\mathcal{T}_{00},\mathcal{T}_{11},N\big]\Big|N\Big)\quad\text{(by the Law of Total Covariance)}$$

$$+\frac{4}{N^2}\left(\sum_{j=1}^{J}\sum_{k,h,k\neq h}\mathbb{C}\big(D_{j,k},D_{j,h}\big|N\big)+\sum_{j,l,j\neq l}\sum_{k=1}^{K_j}\sum_{s=1}^{K_l}\mathbb{C}\big(D_{j,k},D_{l,s}\big|N\big)\right)$$

$$=\frac{4}{N^2}\sum_{j=1}^{J}\sum_{k=1}^{K_j}\left(\frac{|\mathcal{T}_{11}|}{\mathcal{J}}\mathbb{V}(2Y|\mathcal{T}_{11})+\frac{|\mathcal{T}_{00}|}{\mathcal{J}}\mathbb{V}(2Y|\mathcal{T}_{00})+\frac{|\mathcal{T}_{1}|}{\mathcal{J}}\mathbb{V}(Y|\mathcal{T}_{1})+\frac{|\mathcal{T}_{0}|}{\mathcal{J}}\mathbb{V}(Y|\mathcal{T}_{0})\right)$$

$$+\frac{4}{N^2}\sum_{j=1}^{J}\sum_{k=1}^{K_j}\mathbb{V}\left(\frac{|\mathcal{T}_{11}|}{\mathcal{J}}(\mu_{11}-2\mu)+\frac{|\mathcal{T}_{00}|}{\mathcal{J}}(2\mu-\mu_{00})+\frac{|\mathcal{T}_{1}|}{\mathcal{J}}(\mu_1-\mu)+\frac{|\mathcal{T}_{0}|}{\mathcal{J}}(\mu-\mu_0)\right)$$

$$+\frac{4}{N^2}\left(\sum_{j=1}^{J}\sum_{k,h,k\neq h}\mathbb{C}\big(D_{j,k},D_{j,h}\big|N\big)+\sum_{j,l,j\neq l}\sum_{k=1}^{K_j}\sum_{s=1}^{K_l}\mathbb{C}\big(D_{j,k},D_{l,s}\big|N\big)\right)$$

$$=\frac{4}{N^2}\Big(|\mathcal{T}_{11}|\mathbb{V}(2Y|\mathcal{T}_{11})+|\mathcal{T}_{00}|\mathbb{V}(2Y|\mathcal{T}_{00})+|\mathcal{T}_{1}|\mathbb{V}(Y|\mathcal{T}_{1})+|\mathcal{T}_{0}|\mathbb{V}(Y|\mathcal{T}_{0})\Big)\tag{S30}$$

$$+\frac{4}{N^2\mathcal{J}}\mathbb{V}\Big(|\mathcal{T}_{11}|(\mu_{11}-2\mu)+|\mathcal{T}_{00}|(2\mu-\mu_{00})+|\mathcal{T}_{1}|(\mu_1-\mu)+|\mathcal{T}_{0}|(\mu-\mu_0)\Big)\tag{S31}$$

$$+\frac{4}{N^2}\left(\sum_{j=1}^{J}\sum_{k,h,k\neq h}\mathbb{C}\big(D_{j,k},D_{j,h}\big|N\big)+\sum_{j,l,j\neq l}\sum_{k=1}^{K_j}\sum_{s=1}^{K_l}\mathbb{C}\big(D_{j,k},D_{l,s}\big|N\big)\right).\tag{S32}$$

We have the following key observations:

(A) For Eq (S31), when the null hypothesis of no causality is true, $\mu_{11}=\mu_{00}=2\mu$ and $\mu_1=\mu_0=\mu$ so that (S31) equals zero. When the alternative hypothesis is true, (S31) is a variance so it is non-negative.

(B) For Eq (S32), by Part A and B in Appendix A.8, (S32)$=0$ when the null hypothesis of no causality is true, it follows that (S32)≥ 0 when the alternative hypothesis is true.

Therefore, when the null hypothesis that $\delta_{\text{TMT}}=0$ is true, $\mathbb{V}(d^{\text{pc}}_{\text{gTMT}}|N)=$ Eq (S30). In general, $\mathbb{V}(d^{\text{pc}}_{\text{gTMT}}|N)\geq$ Eq (S30). Notice that Eq (S30) is exactly the right hand side of Eq (14) in Theorem 3. This completes the proof of Theorem 3.

B Simulations

B.1 Simulating genotypes

Here we describe a generative process to simulate genotypes in a nuclear family. Both parental genotypes matrices $\boldsymbol{Z}^m$ and $\boldsymbol{Z}^p$ were sampled from a structured population based on a standard admixture model [2, 3] with $K = 4$ admixed populations. We configured this population to have $F_{\mathrm{ST}} = 0.2$ by utilizing the `bnpsd` R package [1, 4]. We simulated the ancestral allele frequencies from the Uniform$(0.1, 0.9)$, which is an option in the `bnpsd` R package. The population structure quantified by coancestry coefficients between pairs of individuals is displayed in Fig B.

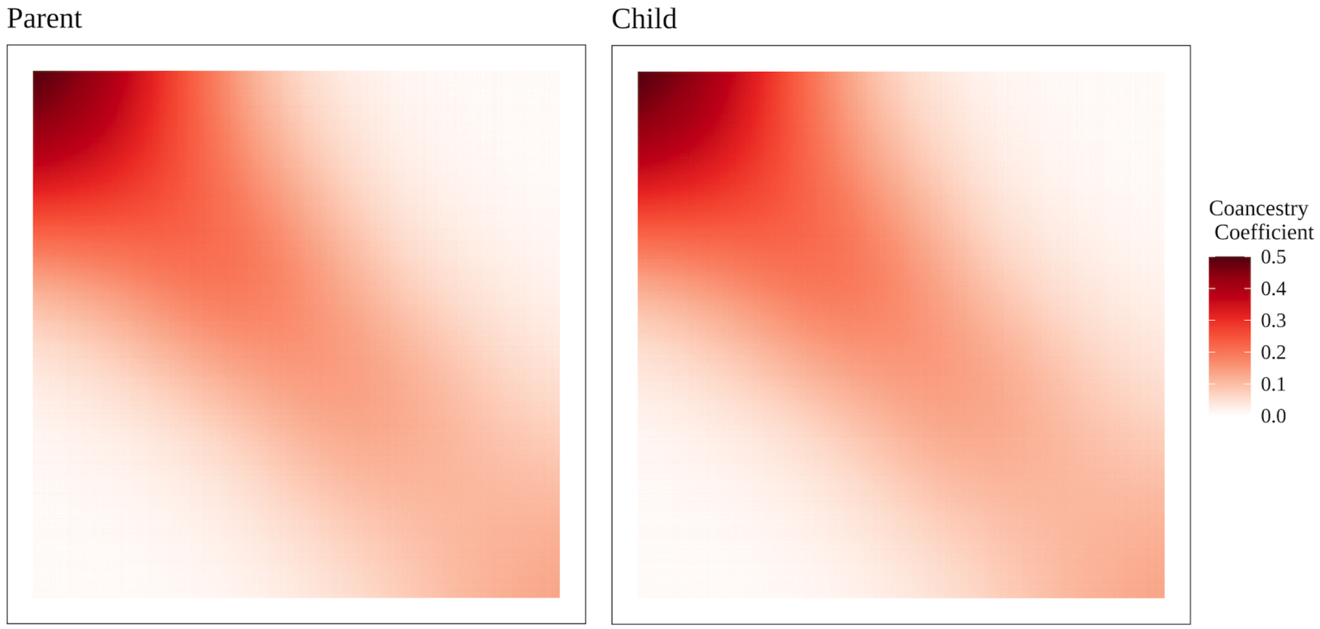

Fig B: **Coancestry coefficients among individuals in the structured population.** (A) Parent coancestry coefficients. (B) Offspring coancestry coefficients. Each entry is a pairwise coancestry coefficient calculated by `popkin` [1] for a simulated sample of 500 trios randomly drawn from the structured population. Simulations utilized in this work with larger sample sizes share the same pattern of population structure and the same variability of coancestry coefficients with an overall $F_{ST} = 0.2$.

B.2 Simulating quantitative traits

We first generated a non-genetic factor associated with the population structure. Let $\boldsymbol{E} = \{E_j\}$ be the random non-genetic factor. We adopted the admixture proportion $\boldsymbol{q} = \{q_{ju}\}$ from Appendix B.1 to simulate E_j as

$$E_j = \sum_{u=1}^{U} q_{ju} R_u, \quad R_u \sim \mathcal{N}(u, 1) \text{ for } u \in [1:U], \quad U = 4.$$

Let $\mathcal{C}$ be the set of causal SNPs. We followed Eq (9) to simulate the child's trait $\boldsymbol{Y} = \{Y_{j,k}\}$ as

$$Y_{j,k} = \iota + \sum_{i \in \mathcal{C}} \Big(\alpha_0 \mathcal{I}(G_{i,j,k} = 0) + \alpha_1 \mathcal{I}(G_{i,j,k} = 1) + \alpha_2 \mathcal{I}(G_{i,j,k} = 2) \Big) + vE_j + \epsilon_{j,k},$$

where we set $\iota = 100$ and simulated $\epsilon_{j,k}$ from Normal(0,σ_e^2) with $\sigma_e^2 = 1$. To show that the above equation satisfies the trait model in Eq (9), at causal SNP c ($c \in \mathcal{C}$), rewrite the equation as

$$\begin{aligned}
Y_{j,k} &= \alpha_0 \mathcal{I}(G_{c,j,k} = 0) + \alpha_1 \mathcal{I}(G_{c,j,k} = 1) + \alpha_2 \mathcal{I}(G_{c,j,k} = 2) + \xi_{j,k} + \gamma_j, \text{ where} \\
\epsilon_{j,k} &= \xi_{j,k} + \sum_{i \in \mathcal{C}, i \neq c} \Big(\alpha_0 \mathcal{I}(G_{i,j,k} = 0) + \alpha_1 \mathcal{I}(G_{i,j,k} = 1) + \alpha_2 \mathcal{I}(G_{i,j,k} = 2) \Big), \\
\gamma_j &= \iota + vE_j.
\end{aligned}$$

Note that the first line satisfies Eq (9). We considered the following parameters

$$\alpha_0 = v, \alpha_1 = 2v, \alpha_2 = 3v.$$

Then the ratio $(\alpha_2 - \alpha_1)/(\alpha_1 - \alpha_0)$ is 1. Given the desired heritability $h^2 = \sigma_a^2/(\sigma_a^2 + \sigma_e^2)$ where we set $\sigma_e^2 = 1$, we simulated v such that $\sigma_a^2 = \mathbb{V}\Big(\iota + \sum_{i \in \mathcal{C}} \alpha_0 \mathcal{I}(G_{i,j,k} = 0) + \sum_{i \in \mathcal{C}} \alpha_1 \mathcal{I}(G_{i,j,k} = 1) + \sum_{i \in \mathcal{C}} \alpha_2 \mathcal{I}(G_{i,j,k} = 2)\Big)$, achieving the desired h^2.

B.3 Numerical data underlying Fig 2B

Table B: **Observed FPR across significant levels.**

(a) The null distribution is Normal(0,1)

	Observed FPR		
α	$h^2 = 0.3$	$h^2 = 0.6$	$h^2 = 0.9$
0.00	0.0000	0.0000	0.0000
0.05	0.0487	0.0494	0.0506
0.10	0.0988	0.1001	0.1012
0.15	0.1493	0.1503	0.1514
0.20	0.2000	0.2003	0.2014
0.25	0.2501	0.2516	0.2514
0.30	0.3014	0.3021	0.3024
0.35	0.3518	0.3518	0.3526
0.40	0.4022	0.4026	0.4020
0.45	0.4535	0.4522	0.4516
0.50	0.5017	0.5013	0.5014
0.55	0.5519	0.5513	0.5512
0.60	0.6017	0.6013	0.6020
0.65	0.6504	0.6504	0.6516
0.70	0.7006	0.7002	0.7019
0.75	0.7499	0.7503	0.7502
0.80	0.8005	0.8005	0.8004
0.85	0.8516	0.8505	0.8491
0.90	0.9010	0.9006	0.8999
0.95	0.9504	0.9495	0.9502
1.00	1.0000	1.0000	1.0000

(b) The null is constructed by permutation

	Observed FPR		
α	$h^2 = 0.3$	$h^2 = 0.6$	$h^2 = 0.9$
0.00	0.0000	0.0000	0.0000
0.05	0.0480	0.0493	0.0512
0.10	0.0973	0.0988	0.1011
0.15	0.1462	0.1485	0.1511
0.20	0.1968	0.1991	0.2010
0.25	0.2465	0.2491	0.2504
0.30	0.2964	0.2994	0.3004
0.35	0.3564	0.3594	0.3603
0.40	0.3975	0.3981	0.3995
0.45	0.4476	0.4475	0.4496
0.50	0.4958	0.4981	0.4982
0.55	0.5453	0.5461	0.5483
0.60	0.5946	0.5950	0.5976
0.65	0.6436	0.6451	0.6466
0.70	0.7025	0.7038	0.7054
0.75	0.7428	0.7438	0.7448
0.80	0.7922	0.7935	0.7934
0.85	0.8418	0.8432	0.8421
0.90	0.8914	0.8917	0.8912
0.95	0.9503	0.9505	0.9507
1.00	0.9901	0.9900	0.9904

B.4 Simulating linkage disequilibrium (LD)

For the parental genotypes, we used the software `msprime` (version 1.0) [5] and followed the American Admixture model [6] to simulate 5,000 pairs of parents from the admixed population with 100,000 SNPs across 22 pairs of chromosomes per individual. Our simulation parameters for `msprime` are the same as parameters listed in the supporting information for [6]. We used the R package `popkin` [1] to calculate the F_{ST} of the parental genotypes, which is around 0.18. We set the total number of SNPs in each chromosome proportional to the corresponding chromosome length in Human Genome Assembly GRCh38.p14. We set the mutation rate per generation so that the allele frequency is between 0.05 and 0.95. We set the recombination rate so that the level of LD matches previous findings in the human genome [7, 8]. We randomly selected 4000 pairs of parents to simulate one child per family, and 1000 pairs of parents to have two offspring per family. Within each family, for each chromosome per offspring, we randomly drew a haplotype per parental side and merge these two haplotypes as the offspring genotypes.

To simulate offspring phenotypes, we first simulated a quantitative trait for the offspring phenotype to implement the gTMT. Among the offspring genotypes $\boldsymbol{G}$, we randomly chose 10 causal SNPs denoted by set $\mathcal{C}$. We generated the child phenotypes $\boldsymbol{Y}$ by the polygenic trait model $Y_j = \iota + \sum_{i \in \mathcal{C}} bG_{ij} + \epsilon_j$ where we drew ϵ_j from Normal$(0, \sigma_e^2)$ and we set $\sigma_e^2 = 1$, $\iota = 100$. The coefficient for causal SNPs, b, is determined such that $\mathbb{V}(\sum_{i \in \mathcal{C}} bG_{ij})/\sigma_e^2 = 1$ (i.e., the heritability $h^2 = 0.5$).

We display the gTMT statistic d_{gTMT} and corresponding p-values across all 100,000 SNPs as the genome-wide TMT profile (Fig CA). To compare with linkage-equilibrium (LE), we permuted trio genotypes to remove linkage disequilibrium, regenerated the child traits using the same parameters in the polygenic trait model above, and conducted the gTMT on a per locus basis to derive d_{gTMT} and p-values (Fig CB). Under both LD and LE scenarios, the gTMT shows accurate ACE estimation at the causal loci. The p-values of gTMT are smaller at the causal loci than at the noncausal loci linked to them.

In Fig DA, we present pair-wise r^2 around a causal locus suggesting different LD strengths. The level of LD in our simulated genotypes (Fig DB) matches previous findings observed in the human genome [7, 8].

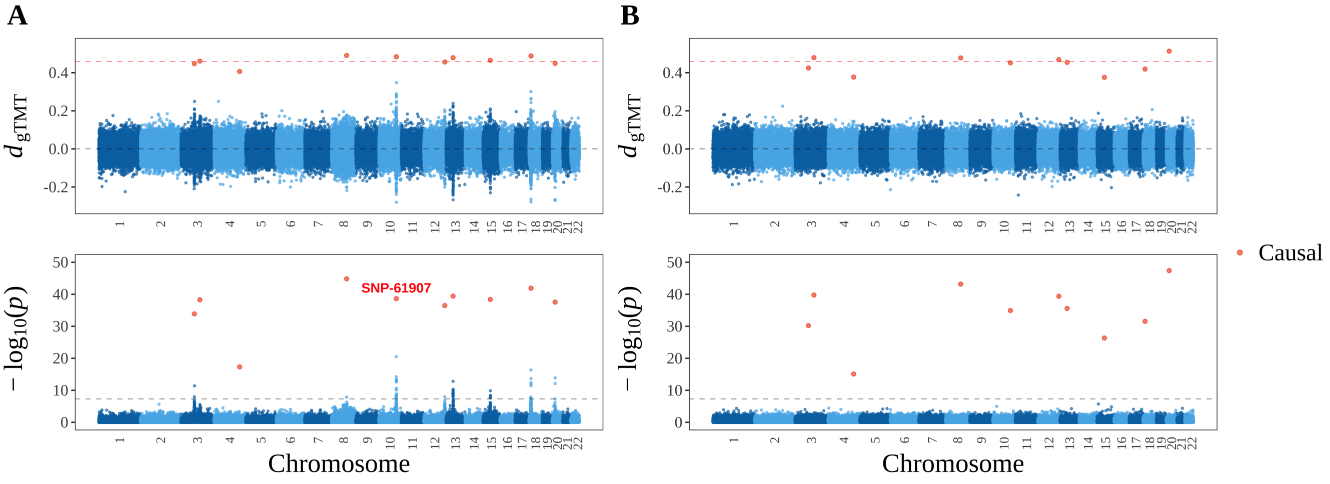

Fig C: **The genome-wide gTMT profile under (A) linkage-disequilibrium (LD) and (B) linkage equilibrium (LE).** The gTMT profile is presented as d_{gTMT} and $-\log_{10}(p)$ at 100,000 SNPs across 22 chromosomes simulated by `msprime`. In the upper panels, the red dashed line is the true ACE at the causal loci and the black dashed line is for zero ACE at the non-causal loci. In the lower panels, the gray dashed line is at p-value $= 5 \times 10^{-8}$, which is commonly used as a p-value threshold in GWAS. In (A), the causal SNP-61907 has its nearby LD pattern shown in Fig D.

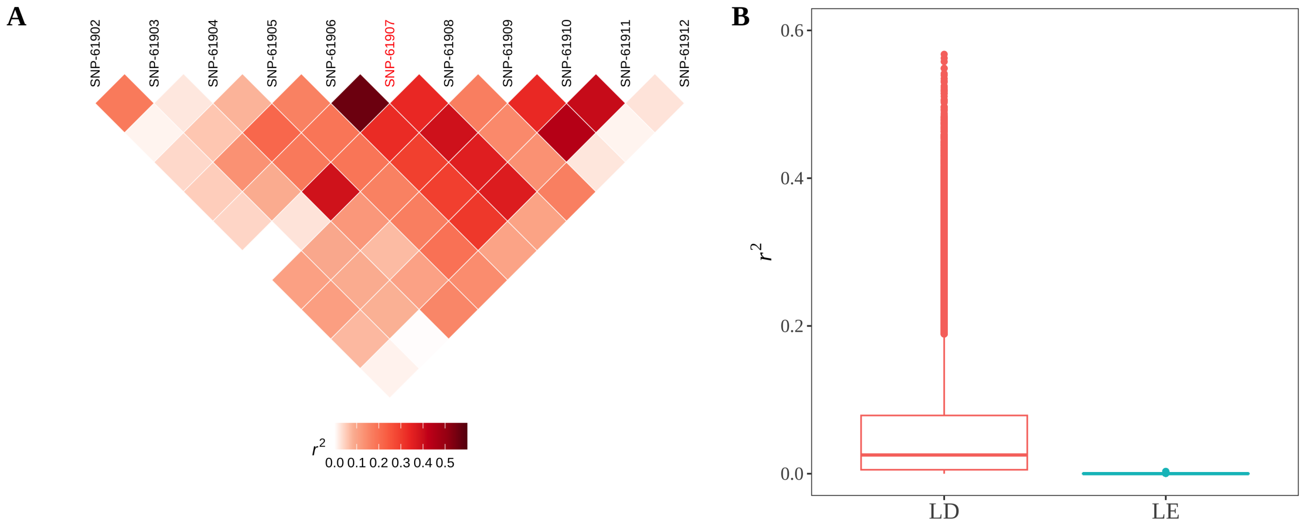

Fig D: **The LD pattern presented as (A) pair-wise r^2 between SNPs near a causal variant (SNP-61907 from Fig C) and (B) the distribution of r^2 between adjacent SNPs across all 100,000 simulated SNPs.** In (B), the r^2 values are shown for all pairs of adjacent SNPs (with the physical distance around 30 kb) for the simulated linkage-disequilibrium (LD) scenario and the linkage-equilibrium (LE) scenario after permutation.

B.5 Simulating family-specific confounding effects

We followed Appendix B.1 to simulate genotypes of 1,500 nuclear families (500 trios, 500 tetrads and 500 quintets). This leads to 3,000 offspring and 3,000 parents with 100,000 SNPs per individual. We randomly chose 100 causal SNPs and denoted them as the causal SNP set $\mathcal{C}$. We randomly chose a target causal locus i^* from $\mathcal{C}$ for testing. Let $\mathcal{I}(\cdot)$ be the indicator function. We generated a family-specific confounding effect κ_{i^*j} that is correlated with the parental homozygous genotypes at locus i by $\kappa_{i^*j} = (Z^m_{i^*j} + Z^p_{i^*j} - \xi)\mathcal{I}(Z^m_{i^*j} \neq 1, Z^p_{i^*j} \neq 1)$. We generated child phenotypes according to

$$Y_{j,k} = \iota + \sum_{c \in \mathcal{C}} bG_{i,j,k} + s\kappa_{i^*j} + \epsilon_{j,k},$$

where we generated $\epsilon_{j,k}$ from Normal$(0, \sigma_e^2)$ and we set $\sigma_e^2 = 1$, $\iota = 100$, $\xi = 10$. The coefficient b is determined such that $\mathbb{V}(\sum_{i \in \mathcal{C}} bG_{i,j,k})/\sigma_e^2 = 9$.

We explored the performances of gTMT, FBAT, and FGWAS under the following three scenarios.

Scenario A: The confounder κ_{i^*j} depends on homozygous parental genotypes in that $\kappa_{i^*j} = (Z^m_{i^*j} + Z^p_{i^*j} - \xi)\mathcal{I}(Z^m_{i^*j} \neq 1, Z^p_{i^*j} \neq 1)$;

Scenario B: The confounder κ_{i^*j} depends on heterozygous parental genotypes in that $\kappa_{i^*j} = (Z^m_{i^*j} + Z^p_{i^*j} - \xi)\mathcal{I}(Z^m_{i^*j} = 1 \text{ or } Z^p_{i^*j} = 1)$;

Scenario C: The confounder κ_{i^*j} is simply a linear combination of parental genotypes in that $\kappa_{i^*j} = Z^m_{i^*j} + Z^p_{i^*j} - \xi$. This setting is most ideal for FGWAS because FGWAS controls confounding effects in this scenario by regression conditional on parental genotypes.

Under each scenario, we followed Appendix B.1 in S1 Text to simulate genotypes of 3000 families (1000 trios, 1000 tetrads, and 1000 quintets), containing 6000 offspring with 100,000 SNPs per individual. We randomly chose 100 causal SNPs (denoted as the set $\mathcal{C}$), simulated confounder κ_{i^*j} at a causal locus i^* randomly drawn from $\mathcal{C}$, and generated the offspring phenotype by $Y_{j,k} = \iota + \sum_{i \in \mathcal{C}} bG_{i,j,k} + s\kappa_{i^*j} + \epsilon_{j,k}$, where we generated $\epsilon_{j,k}$ from Normal$(0, \sigma_e^2)$ and we set $\sigma_e^2 = 1$, $\iota = 100$, $\xi = 8$, and b is determined such that $\mathbb{V}(\sum_{i \in \mathcal{C}} bG_{i,j,k})/\sigma_e^2 = 9$. We applied gTMT, FBAT, and FGWAS to compute p-values at the causal loci. Specifically, for 500 iterations, we generated offspring genotypes at the causal loci, simulated offspring traits, and applied the three methods to derive p-values. This produced 500 p-values per causal locus per method. We calculated empirically the type II error (β) as the proportion of p-values greater or equal to a significance-level $\alpha = 0.05$ among 500 p-values for each method, i.e., $\beta = (\text{no. } p\text{-values} \geq \alpha)/500$. Then we used $1 - \beta$

to calculate the statistical power. The gTMT is robust to confounding effects across all three scenarios, which is true even in Scenario C that may in theory favor FGWAS over gTMT.

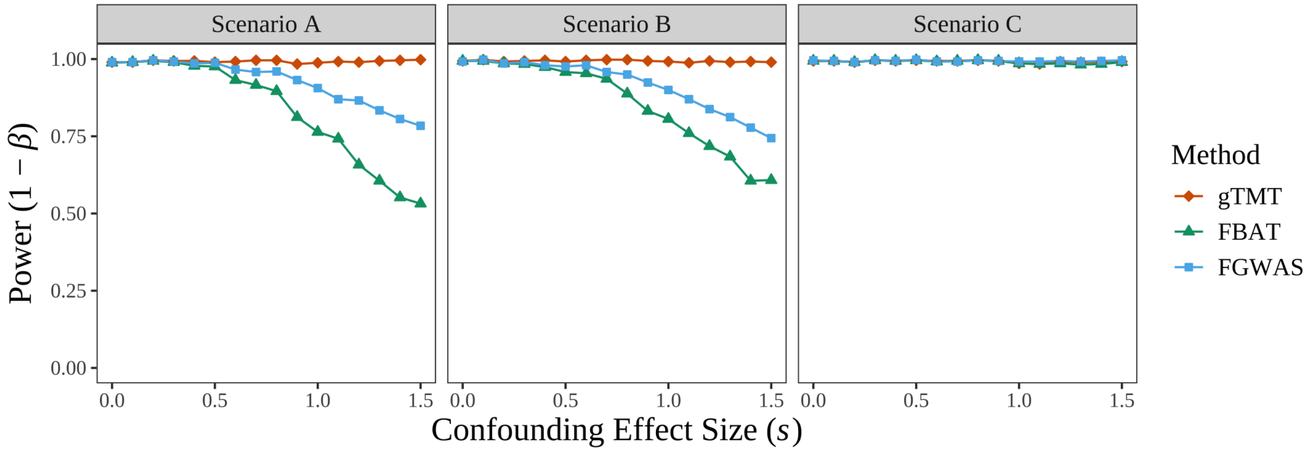

Fig E: **Statistical power for detecting causal effects using gTMT, FBAT, and FGWAS under three scenarios of confounding effects.** In Scenario A, the confounder depends on homozygous parental genotypes. In Scenario B, the confounder depends on heterozygous parental genotypes. In Scenario C, the confounder is a linear combination of parental genotypes.

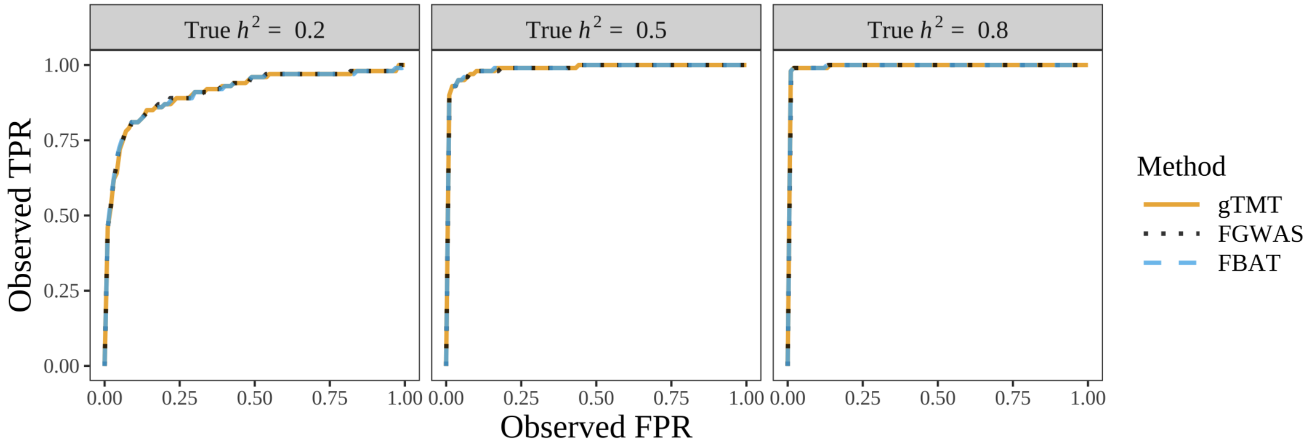

Fig F: **ROC curves for gTMT, FBAT, and FGWAS without confounding effects.**

We compared the statistical power of gTMT, FGWAS, and FBAT in the scenario without confounding.We simulated genotypes of 3000 families (1000 trios, 1000 tetrads, and 1000 quintets) with 6000 offspring, 100,000 SNPs per individual. We randomly chose 100 causal loci (denoted as the set $\mathcal{C}$) and followed the polygenic trait model without confounding effects to simulate offspring phenotype by $Y_{j,k} = \iota + \sum_{i \in \mathcal{C}} bG_{i,j,k} + \epsilon_{j,k}$, where $\iota = 100$, $\epsilon_{j,k} \sim \text{Normal}(0, \sigma_e^2)$ with $\sigma_e^2 = 1$, and b

set to achieve the desired true heritability h^2 in that $\mathbb{V}(\sum_{i\in\mathcal{C}} bG_{i,j,k})/\sigma_e^2 = h^2/(1-h^2)$. We explored different values of h^2 (0.2,0.5,0.8) and implemented gTMT, FGWAS, and FBAT across all SNPs to derive p-values to construct ROC curves. In Fig F, all three methods resulted in similar ROC curves, showing very close statistical power without confounding effects.

In the main manuscript, for κ_{i^*j} depending on homozygous parental genotypes in that $\kappa_{i^*j} = (Z^m_{i^*j} + Z^p_{i^*j} - \xi)\mathcal{I}(Z^m_{i^*j} \neq 1, Z^p_{i^*j} \neq 1)$, we chose $\xi = 10$ which is an arbitrary number. Here, we tried other choices of ξ (3,6,12) to explore the statistical power of gTMT, FGWAS, and FBAT when confounding exists. Given each value of ξ, we followed Appendix B.1 to simulate genotypes of 3000 families (1000 trios, 1000 tetrads, and 1000 quintets), containing 6000 offspring with 100,000 SNPs per individual. We randomly chose 100 causal SNPs (denoted as the set $\mathcal{C}$), simulated κ_{i^*j} at a causal locus i^* randomly drawn from $\mathcal{C}$, and generated offspring phenotypes by $Y_{j,k} = \iota + \sum_{i\in\mathcal{C}} bG_{i,j,k} + s\kappa_{i^*j} + \epsilon_{j,k}$, where we generated $\epsilon_{j,k}$ from the standard normal distribution and set other parameters following Appendix B.5. We applied gTMT, FBAT and FGWAS to compute p-values at the causal loci. Specifically, for 500 iterations, we generated offspring genotypes at the causal loci, simulated offspring traits, and applied the three methods to derive p-values. This produced 500 p-values per causal locus per method. We calculated empirically the type II error (β) as the proportion of p-value greater or equal to a significance-level $\alpha = 0.05$ among 500 p-values for each method, i.e., $\beta = (\text{no. } p\text{-values} \geq \alpha)/500$. Then we used $1 - \beta$ to estimate the statistical power and presented the results in Fig G. Across all levels of ξ, gTMT appears to be more robust to the confounding effects than the other two association methods.

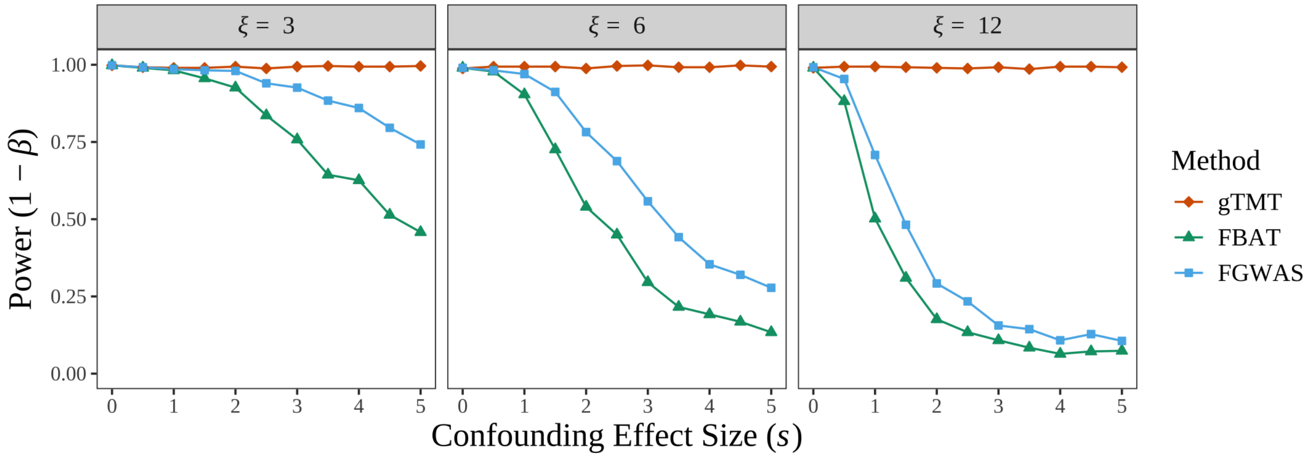

Fig G: **Statistical power for detecting causal effects using gTMT, FBAT, and FGWAS using various values of ξ to simulate confounding effects.**

C UK Biobank data analysis

C.1 Identifying trios

We followed the criteria in KING [9] to identify 28,895 parent–child pair candidates that have estimated kinship around 1/4 (between $2^{-5/2}$ and $2^{-3/2}$) and a discordant homozygote rate less than 0.1. We filtered pairs whose age difference is less than 17, leaving 4,494 mother-child pairs and 1,998 father-child pairs. We excluded four mother-child pairs that share the same child because the mothers are twins. Among the remaining parent–child pairs, we detected nuclear families that have both parents available and at least one child, leading to 990 trios (two parents and one child) and 37 quartets (two parents and two offspring) with 1,064 distinct offspring and 2,054 parents in total.

C.2 Blood pressure phenotypes quality control

We used two original phenotypes in UK Biobank including systolic blood pressure (SBP, field id p4080) and diastolic blood pressure (DBP, field id p4079). These are automated reading results and each phenotype contains two repeated measures per individual that were taken a few moments apart. We calculated the mean of two repeated measures per individual as the target trait. When automated reading results were missing, we used manual reading measures of systolic blood pressure (field id p93) and diastolic blood pressure (field id p94) to fill missing values. Among our study cohort, 1,062 offspring have valid blood pressure records and two offspring have neither automated nor manual reading results. We followed a previous QC pipeline [10] to adjust SBP and DBP for the use of blood pressure lowering medication.

C.3 Top significant variants supported by existing studies

Table C: **Top significant variants and nearest protein coding genes identified by gTMT and supported by existing studies.**

Gene	Chr.	Position	SNP ID	Validation	*P* Value		
					gTMT	FGWAS	FBAT
**Systolic Blood Pressure**							
WRAP73	1	3557213	rs574443061	[11]	3.2×10^{-8}	1.8×10^{-2}	1.3×10^{-3}
PRKCE	2	46040900	rs146431297	[10–20]	5.9×10^{-7}	6.3×10^{-2}	1.9×10^{-4}
C1D	2	68238507	rs116280779	[18]	8.4×10^{-7}	1.1×10^{-2}	2.1×10^{-3}
PLXNA1	3	126743439	rs138755906	[21]	3.6×10^{-8}	4.5×10^{-6}	1.2×10^{-2}
ROBO1	3	78563872	rs112301420	[14, 16, 18, 20]	8.9×10^{-7}	1.4×10^{-4}	2.7×10^{-4}
ROBO1	3	78606526	rs113809588	[14, 16, 18, 20]	3.7×10^{-7}	1.3×10^{-4}	4.1×10^{-4}
PAM	5	102247916	rs72783884	[22, 23]	8.3×10^{-9}	4.5×10^{-2}	5.3×10^{-3}
PDE10A	6	166419485	rs300136	[12, 14–20, 24, 25]	3.6×10^{-7}	2.1×10^{-3}	2.6×10^{-4}
CUX1	7	101826545	rs116903233	[11]	1.0×10^{-7}	2.8×10^{-6}	9.7×10^{-7}
CUX1	7	101834007	rs117264186	[11]	3.9×10^{-7}	3.6×10^{-6}	2.7×10^{-6}
SOX6	11	15844310	rs12421307	[11–20, 24–33]	5.1×10^{-7}	3.6×10^{-3}	1.7×10^{-3}
GPC6	13	94427053	rs6492679	[11, 16, 34, 35]	9.1×10^{-8}	1.8×10^{-4}	1.0×10^{-3}
TMEM100	17	53735407	rs7220190	[13]	2.0×10^{-8}	1.7×10^{-5}	2.5×10^{-5}
**Diastolic Blood Pressure**							
SLC39A10	2	195335316	rs78350181	[15, 16]	1.6×10^{-8}	2.0×10^{-2}	4.0×10^{-4}
FOXP1	3	70786977	rs73114142	[16, 17, 20]	1.7×10^{-7}	5.3×10^{-4}	1.9×10^{-4}
ZNF777	7	149156087	rs147316625	[15–17, 20, 36]	8.1×10^{-7}	4.6×10^{-4}	3.2×10^{-5}
VLDLR	9	2610715	rs76322239	[16, 17, 37, 38]	6.0×10^{-8}	2.2×10^{-1}	7.1×10^{-4}
CAMK1D	10	12459806	rs138764728	[39]	5.2×10^{-7}	9.7×10^{-5}	2.0×10^{-5}
HMGA2	12	66401766	rs77810967	[11]	2.5×10^{-7}	1.4×10^{-3}	1.8×10^{-4}
CRADD	12	94344561	rs74429726	[16]	9.1×10^{-8}	7.4×10^{-5}	8.7×10^{-5}
WDR7	18	54736614	rs573195412	[16, 17]	7.9×10^{-7}	1.3×10^{-2}	1.5×10^{-4}
**Pulse Pressure**							
WRAP73	1	3557213	rs574443061	[11]	6.1×10^{-8}	1.5×10^{-2}	8.8×10^{-4}
CAPG	2	85639364	rs189750681	[17]	1.5×10^{-7}	3.8×10^{-2}	3.7×10^{-4}
HRH1	3	11184774	rs115199870	[12]	8.2×10^{-7}	4.9×10^{-5}	9.8×10^{-4}
IRX	5	2382979	rs458479	[40]	2.6×10^{-7}	1.5×10^{-3}	3.9×10^{-6}
SLC20A2	8	42286501	rs74820353	[10, 12, 13, 15, 16, 32]	1.3×10^{-7}	3.9×10^{-2}	8.6×10^{-6}
REEP3	10	66012934	rs72837596	[12, 16, 19]	9.3×10^{-7}	5.8×10^{-3}	7.2×10^{-4}
FARP1	13	98977986	rs118149456	[11, 16]	3.1×10^{-7}	4.1×10^{-2}	8.0×10^{-4}

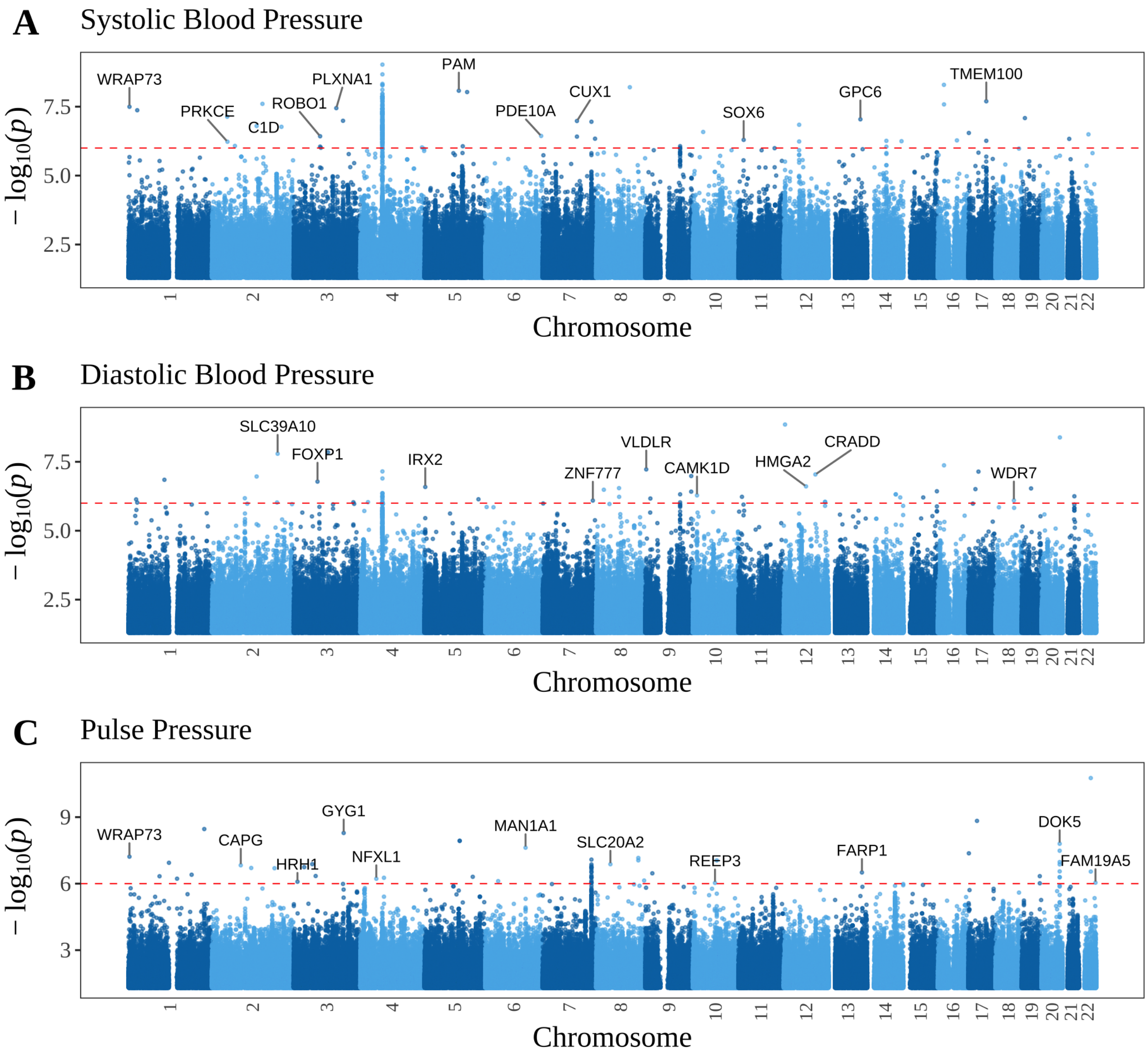

Fig H: **UK Biobank genome-wide gTMT profile for blood pressure measures including (A) systolic blood pressure; (B) diastolic blood pressure; (C) pulse pressure.** The red dashed line is for p-value$= 1 \times 10^{-6}$.

References

[1] Alejandro Ochoa and John D Storey. "Estimating FST and kinship for arbitrary population structures". *PLoS Genetics* 17(1) (2021), e1009241.

[2] David H Alexander, John Novembre, and Kenneth Lange. "Fast model-based estimation of ancestry in unrelated individuals". *Genome Research* 19(9) (2009), pp. 1655–1664.

[3] Irineo Cabreros and John D Storey. "A likelihood-free estimator of population structure bridging admixture models and principal components analysis". *Genetics* 212(4) (2019), pp. 1009–1029.

[4] Alejandro Ochoa and John D Storey. *Package 'bnpsd'*. R package version 1.3.13. 2021.

[5] Franz Baumdicker et al. "Efficient ancestry and mutation simulation with msprime 1.0". *Genetics* 220(3) (2022), iyab229.

[6] Sharon R Browning et al. "Ancestry-specific recent effective population size in the Americas". *PLoS Genetics* 14(5) (2018), e1007385.

[7] David E Reich et al. "Linkage disequilibrium in the human genome". *Nature* 411(6834) (2001), pp. 199–204.

[8] Elisabeth Dawson et al. "A first-generation linkage disequilibrium map of human chromosome 22". *Nature* 418(6897) (2002), pp. 544–548.

[9] Ani Manichaikul et al. "Robust relationship inference in genome-wide association studies". *Bioinformatics* 26(22) (2010), pp. 2867–2873.

[10] Helen R Warren et al. "Genome-wide association analysis identifies novel blood pressure loci and offers biological insights into cardiovascular risk". *Nature Genetics* 49(3) (2017), pp. 403–415.

[11] Xiaofeng Zhu et al. "Genome-wide pleiotropy analysis identifies novel blood pressure variants and improves its polygenic risk scores". *Genetic Epidemiology* 46(2) (2022), pp. 105–121.

[12] Thomas J Hoffmann et al. "Genome-wide association analyses using electronic health records identify new loci influencing blood pressure variation". *Nature Genetics* 49(1) (2017), pp. 54–64.

[13] Ayush Giri et al. "Trans-ethnic association study of blood pressure determinants in over 750,000 individuals". *Nature Genetics* 51(1) (2019), pp. 51–62.

[14] Gleb Kichaev et al. "Leveraging polygenic functional enrichment to improve GWAS power". *American Journal of Human Genetics* 104(1) (2019), pp. 65–75.

[15] Saori Sakaue et al. "A cross-population atlas of genetic associations for 220 human phenotypes". *Nature Genetics* 53(10) (2021), pp. 1415–1424.

[16] Jacob M Keaton et al. "Genome-wide analysis in over 1 million individuals of European ancestry yields improved polygenic risk scores for blood pressure traits". *Nature Genetics* 56(5) (2024), pp. 778–791.

[17] Fotios Koskeridis et al. "Multi-trait association analysis reveals shared genetic loci between Alzheimer's disease and cardiovascular traits". *Nature Communications* 15(1) (2024), p. 9827.

[18] Sinan Shi et al. "A Genomics England haplotype reference panel and imputation of UK Biobank". *Nature Genetics* 56(9) (2024), pp. 1800–1803.

[19] Anurag Verma et al. "Diversity and scale: Genetic architecture of 2068 traits in the VA Million Veteran Program". *Science* 385(6706) (2024), eadj1182.

[20] Yon Ho Jee et al. "Genome-wide association studies in a large Korean cohort identify quantitative trait loci for 36 traits and illuminate their genetic architectures". *Nature Communications* 16(1) (2025), p. 4935.

[21] Swaib A Lule et al. "A genome-wide association and replication study of blood pressure in Ugandan early adolescents". *Molecular Genetics & Genomic Medicine* 7(10) (2019), e00950.

[22] Minjoo Kim et al. "EPHA6 rs4857055 C $>$ T polymorphism associates with hypertension through triglyceride and LDL particle size in the Korean population". *Lipids in Health and Disease* 16(1) (2017), p. 230.

[23] Pavithra Nagarajan et al. "A large-scale genome-wide study of gene-sleep duration interactions for blood pressure in 811,405 individuals from diverse populations". *Molecular Psychiatry* 30(8) (2025), pp. 3660–3672.

[24] Tanika N Kelly et al. "Insights from a large-scale whole-genome sequencing study of systolic blood pressure, diastolic blood pressure, and hypertension". *Hypertension* 79(8) (2022), pp. 1656–1667.

[25] Alfred Pozarickij et al. "Causal relevance of different blood pressure traits on risk of cardiovascular diseases: GWAS and Mendelian randomisation in 100,000 Chinese adults". *Nature Communications* 15(1) (2024), p. 6265.

[26] Nora Franceschini et al. "Genome-wide association analysis of blood-pressure traits in African-ancestry individuals reveals common associated genes in African and non-African populations". *American Journal of Human Genetics* 93(3) (2013), pp. 545–554.

[27] Xiangfeng Lu et al. "Genome-wide association study in Chinese identifies novel loci for blood pressure and hypertension". *Human Molecular Genetics* 24(3) (2015), pp. 865–874.

[28] Georg B Ehret et al. "The genetics of blood pressure regulation and its target organs from association studies in 342,415 individuals". *Nature Genetics* 48(10) (2016), pp. 1171–1184.

[29] Yun J Sung et al. "A large-scale multi-ancestry genome-wide study accounting for smoking behavior identifies multiple significant loci for blood pressure". *American Journal of Human Genetics* 102(3) (2018), pp. 375–400.

[30] Fumihiko Takeuchi et al. "Interethnic analyses of blood pressure loci in populations of East Asian and European descent". *Nature Communications* 9(1) (2018), p. 5052.

[31] Daokun Sun et al. "Multi-ancestry genome-wide association study accounting for gene-psychosocial factor interactions identifies novel loci for blood pressure traits". *Human Genetics and Genomics Advances* 2(1) (2021).

[32] Min-Lee Yang et al. "Sex-specific genetic architecture of blood pressure". *Nature Medicine* 30(3) (2024), pp. 818–828.

[33] Hrushikesh Loya et al. "A scalable variational inference approach for increased mixed-model association power". *Nature Genetics* 57(2) (2025), pp. 461–468.

[34] Daniel Levy et al. "Framingham Heart Study 100K Project: genome-wide associations for blood pressure and arterial stiffness". *BMC Medical Genetics* 8(Suppl 1) (2007), S3.

[35] Yun J Sung et al. "Gene–smoking interactions identify several novel blood pressure loci in the Framingham heart study". *American Journal of Hypertension* 28(3) (2015), pp. 343–354.

[36] Praveen Surendran et al. "Discovery of rare variants associated with blood pressure regulation through meta-analysis of 1.3 million individuals". *Nature Genetics* 52(12) (2020), pp. 1314–1332.

[37] Evangelos Evangelou et al. "Genetic analysis of over 1 million people identifies 535 new loci associated with blood pressure traits". *Nature Genetics* 50(10) (2018), pp. 1412–1425.

[38] Jenni M Rimpelä et al. "Genome-wide association study of white-coat effect in hypertensive patients". *Blood Pressure* 28(4) (2019), pp. 239–249.

[39] Francesca Frau et al. "Genome-wide association study identifies CAMKID variants involved in blood pressure response to losartan: the SOPHIA study". *Pharmacogenomics* 15(13) (2014), pp. 1643–1652.

[40] Brenda Udosen et al. "Meta-analysis and multivariate GWAS analyses in 80,950 individuals of African ancestry identify novel variants associated with blood pressure traits". *International Journal of Molecular Sciences* 24(3) (2023), p. 2164.
